# Anticancer function of microRNA‐30e is mediated by negative regulation of *HELLPAR* , a noncoding macroRNA, and genes involved in ubiquitination and cell cycle progression in prostate cancer

**DOI:** 10.1002/1878-0261.13255

**Published:** 2022-06-14

**Authors:** Kavya Ganapathy, Christopher Ngo, Thomas Andl, Domenico Coppola, Jong Park, Ratna Chakrabarti

**Affiliations:** ^1^ Burnett School of Biomedical Sciences University of Central Florida Orlando Florida USA; ^2^ Department of Pathology Moffitt Cancer Center Tampa Florida USA; ^3^ Florida Digestive Health Specialists Bradenton Florida USA; ^4^ Department of Cancer Epidemiology Moffitt Cancer Center Tampa Florida USA

**Keywords:** apoptosis, cell cycle, *HELLPAR*, microRNA, transcriptomics, ubiquitination

## Abstract

Prostate cancer (PCa) progression relies on androgen receptor (AR) function, making AR a top candidate for PCa therapy. However, development of drug resistance is common, which eventually leads to development of castration‐resistant PCa. This warrants a better understanding of the pathophysiology of PCa that facilitates the aberrant activation of key signaling pathways including AR. MicroRNAs (miRNAs) function as regulators of cancer progression as they modulate various cellular processes. Here, we demonstrate a multidimensional function of miR‐30e through the regulation of genes involved in various signaling pathways. We noted loss of miR‐30e expression in prostate tumors, which, when restored, led to cell cycle arrest, induction of apoptosis, improved drug sensitivity of PCa cells and reduced tumor progression in xenograft models. We show that experimental upregulation of miR‐30e reduces expression of mRNAs including AR, FBXO45, SRSF7 and MYBL2 and a novel long noncoding RNA (lncRNA) *HELLPAR,* which are involved in cell cycle, apoptosis and ubiquitination, and the effects could be rescued by inhibition of miR‐30e expression. RNA immunoprecipitation analysis confirmed direct interactions between miR‐30e and its RNA targets. We noted a newly identified reciprocal relationship between miR‐30e and *HELLPAR,* as inhibition of *HELLPAR* improved stabilization of miR‐30e. Transcriptome profiling and quantitative real‐time PCR (qRT‐PCR) validation of miR‐30e‐expressing PCa cells showed differential expression of genes involved in cell cycle progression, apoptosis and ubiquitination, which supports our *in vitro* study. This study demonstrates an integrated function of miR‐30e on dysregulation of miRNA/lncRNA/mRNA axes that may have diagnostic and therapeutic significance in aggressive PCa.

## Graphical abstract

This study describes a tumor suppressor role of microRNA miR‐30e in prostate cancer (PCa), mediated by modulation of AR signaling, cell cycle, apoptosis and ubiquitination pathways. This is achieved by regulating direct mRNA effectors (AR, FBXO45, SRSF7, and MYBL2), indirect mRNA effectors, and a novel long noncoding RNA (lncRNA) *HELLPAR*. The function of miR‐30e impacts PCa progression through a microRNA‐mRNA‐lncRNA regulatory network.

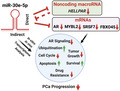

**Abbreviations**
ABI: abiraterone
AR: androgen receptor
BP: biological process
CC: cellular component
ceRNA: competitive endogenous RNA
CRPC: castration‐resistant prostate cancer
DOX: doxycycline
DTX: docetaxel
ENZ: enzalutamide
GO: gene ontology
LncRNAs: long noncoding RNAs
MF: molecular function
miRNAs: microRNAs
PCa: prostate cancer
qPCR: quantitative real‐time PCR
RNA‐seq: RNA sequencing
siRNA: small interfering RNA
TCGA: The Cancer Genome Atlas

## 1 Introduction

MicroRNAs (miRNAs) are involved in gene silencing through inhibition of translation and destabilization of mRNAs, and thereby regulation of a variety of signaling pathways [1, 2]. Dysregulated expression of miRNAs contributes to the abnormal expression of mRNAs which mediates phenotypic changes in various diseases including cancer [3]. The phenomenon that a single miRNA can target multiple mRNAs involved in various biological processes, such as cell proliferation, cell survival, apoptosis and development of drug resistance [2], led to multiple studies focused on the utility of miRNAs as prognostic markers and therapeutic targets for advanced cancer [4, 5]. Since miRNAs can modulate functions of multiple genes and different signaling pathways simultaneously, they offer a therapeutic advantage to thwart development of resistance to monotherapies [2]. miRNAs also interact with long noncoding RNAs (lncRNAs), a group of emerging regulatory molecules that gained attention as potential biomarkers and therapeutic targets for different diseases including cancer [6]. LncRNAs also regulate various molecular processes through direct and indirect interactions with DNA, RNA and proteins and often function as competitive endogenous RNAs (ceRNAs) that specifically bind to miRNAs and prevent their binding to target mRNAs [7]. A number of studies showed the involvement of lncRNAs in progression of cancers including prostate cancer.

Prostate cancer (PCa) still claims a significant number of lives of men worldwide [8, 9, 10]. One of the important biological mechanisms associated with PCa aggressiveness is hyperactivation of androgen receptor (AR) signaling pathway [9], and as a result, androgen deprivation therapy (ADT) remains the front line therapy of PCa; however, the effect of ADT is temporary as development of castration‐resistant PCa (CRPC) is commonly noted [11]. However, AR still remained an attractive therapeutic target as most of the CRPC express functional AR [12]. Studies have shown the involvement of different miRNAs that target AR signaling in PCa. The miR‐182/‐183/‐96 cluster driven by AR signaling contributes to PCa progression. miR‐197 and ‐361‐3p are also frequently overexpressed in CRPC [13]. Let‐7 and miR‐299‐3p, on the other hand, function as tumor suppressor miRNAs that directly target AR and are frequently downregulated in aggressive PCa [14]. Other signaling pathways that are commonly targeted in PCa include VEGFA/VEGFR2 axis in CRPC by miR‐299‐3p [14], Wnt/EZH2/miR‐708 signaling to inhibit neuroendocrine differentiation in PCa [15], WWP1 E3 Ubiquitin Ligase targeted by miR‐452 to regulate PCa oncogenesis [16], miR‐16‐5p targeting of AKT3 to regulate cell cycle and apoptosis in PCa [17] and targeting of RhoGTPase/cytoskeleton reorganization/cell cycle pathways by miR‐17‐92a cluster in PCa [18].

A number of lncRNAs have also been reported to be involved in PCa progression. HOTAIR, an oncogenic lncRNA, drives PCa progression, by activation of AR in an androgen‐independent manner in CRPC [19]. We recently showed *PAINT* to be an oncogenic lncRNA that drives PCa progression through altered expression of genes involved in epithelial‐mesenchymal transition and apoptosis [20]. MEG3, a tumor suppressor lncRNA in PCa, activates p53 signaling and reduces the expression of cell cycle markers [21]. Involvement of miRNA/lncRNA/mRNA regulatory networks has been noted in PCa progression. CCAT1, a lncRNA upregulated in CRPC, binds to the tumor suppressor miR‐28‐5p to diminish its anticancer effect in PCa. MEG3 also modulates the activation of the miR‐9‐5p/QKI‐5 signaling axis to inhibit PCa progression [22].

In this study, we show the anticancer function of miR‐30e, a member of the miR‐30 family, through modulation of mRNA expression of genes involved in multiple signaling pathways in PCa. The miR‐30 family consists of miRs‐30a/‐30b/‐30c/‐30d/‐30e that share the same seed sequence but are located on 6q133 (miR‐30a), 8q24.22 (miR‐30b and ‐30d) and 1p34.2 (miR‐30c and miR‐30e) [23]. Studies have shown the tumor suppressor role of miR‐30 family in many cancers, of which miR‐30a is the most studied and well characterized [24, 25, 26, 27]. Here we present studies demonstrating the involvement of novel miRNA/mRNA axes in the tumor suppressor function of miR‐30e through regulation of expression of four direct targets, *AR, FBXO45, SRSF7* and *MYBL2*, and a distinct set of genes, identified by transcriptome analysis, involved in ubiquitination, cell cycle and apoptosis. Additionally, we show a direct interaction but a reciprocal relationship of a novel extremely lncRNA or macroRNA *HELLPAR*, discovered in our RNA‐seq analysis, with miR‐30e.

## 2 Materials and methods

### 2.1 Patient tissues acquisition, ethics approval and participation consent

Prostate tissues collected at the University of Alabama at Birmingham (UAB) were obtained from the Cooperative Human Tissue Network (Southern division) following an Institutional Review Board (IRB) approved protocol and after obtaining informed consent from donors. Patient tissues for *HELLPAR* expression analysis were obtained from Moffitt Cancer Center (MCC) following an IRB‐approved protocol (IRB#: CR3‐105514) and after obtaining informed and written consent from each donor. Ethics approval and consent to participate were obtained from Cooperative Human Tissue Network (Southern division) at the UAB in accordance with an approved IRB protocol. Samples at the MCC were collected in accordance with approved IRB protocol. The study methodologies conformed to the standards set by the Declaration of Helsinki and were approved by the local ethics committee. Formalin‐fixed paraffin‐embedded (FFPE) prostate tissues were macro‐dissected to separate tumor areas and uninvolved areas at UAB and MCC and used for RNA extraction followed by qRT‐PCR for the expression of miR‐30e and *HELLPAR* RNA in patient tissues as described previously [18].

### 2.2 Quantitative real‐time PCR

RNA extraction, cDNA synthesis and qRT‐PCR were performed as described in [14]. Briefly, total RNA was extracted from PCa patient tissues and cell lines using the Direct‐zol RNA extraction Kit (Zymo Research, Irvine, CA, USA) as per manufacturer's protocol. cDNA synthesis was performed for miR‐30e using miScript RT II Kit (Qiagen, Germantown, MD, USA) and for all mRNAs and macroRNA using High Capacity cDNA synthesis kit (Thermo Fisher Scientific). qRT‐PCR was performed using miR‐30e specific primers and three internal small nuclear RNA (snRNA) controls (Systems Biosciences Inc., Palo Alto, CA, USA) and POWER‐UP SYBR green PCR reagent (Thermo Fisher Scientific, Waltham, MA, USA). For all mRNAs, specific primers were used along with EIF3D and RPL13a as internal controls (10X QuantiTect primers, Qiagen) and QuantiTect SYBR PCR Kit (Qiagen). For *HELLPAR* macroRNA qPCR, 10 μm specific forward and reverse primers were used with the human Tata Binding Protein (TBP) as the internal control gene along with GoTaq qPCR Master mix (Promega, Madison, WI, USA). The primer sequences have been provided below for the *HELLPAR* primers [28] and TBP [29]. qRT‐PCR was performed using QuantStudio 7 thermal cycler (Applied Biosystems, Bedford, MA, USA). The Ct values based on SYBR green fluorescence were normalized to the passive reference ROX dye, and the data were analyzed using the $2^{\Delta \Delta C_{t}}$ method after normalization with internal controls.

*HELLPAR* qRT‐PCR Primer 1: F1: GAAGCAGATGTCACGTACGG; R1: CTGACCTCAATGGTATG GAT. *HELLPAR* qRT‐PCR Primer 2: F2: ACACCATTTTGAGTATCATAA; R2: CAGTCAATCTGATA AGAGAG. Human TBP Primer Sequence: F: GGAGAGTTCTGGGATTGTA; R: CTTATCCTCAT GATTACCGCAG.

### 2.3 Western blotting

Total proteins were extracted from C4‐2B‐30e, 22Rv‐1‐30e and PC‐3‐30e stable sublines at 24 and 48 h post induction with doxycycline using RIPA lysis buffer supplemented with phosphatase and protease inhibitors (Fisher Scientific). 50 μg of extracted proteins were separated on a 10% Bis‐Tris gel, transferred to activated PVDF membranes and were probed with primary antibodies specific to AR, PARP, cleaved caspase‐3 and CCND1 (Cell Signaling Technology) and PCNA (Santa Cruz Biotechnology, Santa Cruz, CA, USA). Alpha‐tubulin (Cell Signaling Technology, Danvers, MA, USA) and GAPDH (Sigma Aldrich, St. Louis, MO, USA) antibodies were used as internal controls. ECL Chemiluminescence substrate (BioRad, Hercules, CA, USA) was used to develop the blots which were imaged using a ChemiDoc MP Imaging System (BioRad). Comparative expression was performed based on densitometry analysis using imagej [30] normalized to internal controls.

### 2.4 Cell line maintenance and transfection

PC‐3 cells (RRID: CVCL0035, ATCC) were maintained in F‐12 Kaighn's Modification HAM medium (Sigma Aldrich). C4‐2B (RRID: CVCL4784; a gift from Dr. Leland Chung, Cedars‐Sinai, Los Angeles, CA, USA) were maintained in RPMI‐1640 media (Sigma Aldrich), LNCaP‐104S (RRID: CVCL_M126; a gift from Dr. Shutsung Liao from University of Chicago Medical Center) were maintained in DMEM media (Sigma Aldrich), 22Rv‐1 (RRID: CVCL_1045; ATCC) were maintained in RPMI‐1640 media (Sigma Aldrich) and MDA‐PCa‐2b cells (RRID: CVCL_4748; ATCC) were maintained in F‐12 Kaighn's Modification HAM medium (Sigma Aldrich). All cell lines were cultured in their respective base media supplemented with 10% heat‐inactivated Fetal Bovine Serum (FBS) (Atlanta Biologicals Inc., Lawrenceville, GA, USA) and 1% antibiotic/antimycotic (Life Technologies, Carlsbad, CA, USA). For LNCaP‐104S, the complete working media was further supplemented with 1 ng·mL^−1^ Dihydrotestosterone (DHT), and for MDA‐PCa‐2b cells, the complete working media was supplemented with 25 ng·mL^−1^ cholera toxin, 10 ng·mL^−1^ mouse EGF, 0.005 mm phosphoethanolamine, 100 pg·mL^−1^ hydrocortisone, 45 nm sodium selenite, 0.005 mg·mL^−1^ human recombinant insulin. All cell lines have been authenticated using short tandem repeat profiling within the last 3 years and tested for mycoplasma contamination by DAPI staining. All experiments were performed with mycoplasma‐free cells.

For generation of stable sublines, a doxycycline‐inducible construct containing human miR‐30e precursor sequence (MI0000749) was cloned into the pLVX‐TetOne‐Puro vector (Clontech Laboratories, Mountain View, CA, USA) at the BamH1 site, which contains a ZsGreen fluorescent marker between BamHI and EcoRI restriction sites. C4‐2B, 22Rv‐1 and PC‐3 cells were transfected with pLVX‐TetOne‐miR‐30e using Lipofectamine 3000 transfection reagent (Thermo Fisher Scientific, Waltham, MA, USA), and stable cells were selected by puromycin. Next, cells were induced with doxycycline (1 μg·mL^−1^), and ZsGreen‐positive cells were sorted in a cell sorter (FACS Aria, BD Biosciences, Franklin Lake, NJ, USA) to generate C4‐2B‐30e, 22Rv‐1‐30e and PC‐3‐30e stable cell lines. Overexpression of miR‐30e in each stable subline was confirmed by qRT‐PCR. For all experiments, doxycycline‐induced stable sublines were used along with uninduced respective stable cell lines as the controls. For *in vivo* xenograft experiments, inducible 22Rv‐1‐30e cell line was used to generate tumors with and without doxycycline induction. For knockdown experiments, 22Rv‐1‐30e cell lines were induced with doxycycline, and miR‐30e expression was confirmed by ZsGreen‐positive cells. Induced cells were transfected with miR‐30e inhibitor (miRIDIAN microRNA hairpin inhibitor, MIMAT0000692, Horizon Discovery, Cat# IH‐300661‐08‐0005) or negative control inhibitor (miRIDIAN negative control hairpin, Horizon Discovery, Boyertown, PA, USA) using RNAiMax (Thermo Fisher Scientific). The miR‐30e inhibitor was designed by the supplier using the mature sequence (accession MIMAT0000692) and a proprietary method [31]. Cells were harvested at 48 h post transfection with the inhibitors and used for subsequent experiments. For *HELLPAR* knockdown experiments, 22Rv‐1 cells were transfected with equal mixture of four siRNAs (20 μm; Horizon Discovery) or negative control nontargeting siRNA pool (Horizon Discovery) using RNAiMax (Thermo Fisher Scientific). The *HELLPAR*‐siRNA sequences are provided below.

*HELLPAR* siRNA1: 5’‐GAATATAAGTCTTGGGAA‐3’.
*HELLPAR* siRNA2: 5’‐GGAAAGAAGTCCATATAT‐3’.
*HELLPAR* siRNA3: 5’‐GGTCAGAGGTGGAGATAT‐3’.
*HELLPAR* siRNA4: 5’‐GCATAGAATTTAAGAGCAA‐3’.

### 2.5 Cell cycle and apoptosis

C4‐2B‐30e and 22Rv‐1‐30e stable cell lines were used for cell cycle analysis with or without induction using Propidium Iodide (PI) staining. Cells were prepared by harvesting at 72 h post induction with doxycycline, and the PI staining and flow cytometry were performed as described before [14]. For the apoptosis assay, annexin V staining was performed using a PE Annexin V Apoptosis Detection kit (BD Biosciences, Franklin Lake, NJ, USA) following the manufacturer's protocol using C4‐2B‐30e and 22Rv‐1‐30e stable lines with and without induction as detailed in [14]. Annexin V staining was performed by harvesting cells 24 h after induction with doxycycline. Fluorescent cells were analyzed in Cytoflex S (Beckman Coulter, Miami, FL, USA) Flow Cytometer. Cell cycle analysis was performed using flowjo software (BD biosciences).

### 2.6 Drug sensitivity assay

C4‐2B‐30e, 22Rv1‐30e and PC‐3‐30e stable lines were used with or without induction. All cells were maintained and seeded in charcoal‐stripped FBS (CS‐FBS) containing complete growth medium. For induction, cells were induced with doxycycline (1 μg·mL^−1^) 24 h post seeding or left uninduced to serve as controls. Post 24 h with or without induction, cells were treated with different doses of Enzalutamide (ENZ), Abiraterone (ABI) or Docetaxel (DTX) and were analyzed for cell viability after 72 h using the MTS‐based Cell Titer Aqueous One cell proliferation assay kit (Promega) as per kit protocol.

### 2.7 RNA immunoprecipitation assay

The potential top target mRNAs of miR‐30e were predicted using online databases TargetScan (http://www.targetscan.org/), miRDB (http://mirdb.org/) and miRTar (https://mirtarbase.cuhk.edu.cn/~miRTarBase/miRTarBase_2022/php/index.php), and targets that were present in all three databases were used for further analysis. miR‐30e targets were validated by RNA pulldown using a biotinylated mimic of miR‐30e (3’‐Biotin) or negative control biotinylated mimics (Qiagen) as described in [32].

Briefly, 22Rv‐1 parental cells were transfected with 50 nm of biotinylated‐miR‐30e mimic or biotinylated‐negative control mimic (Qiagen) using RNAiMax (Thermo Fisher Scientific) in a 10‐cm plate per transfection. 48 h post transfection, cells were lysed in the Lysis buffer [20 mm Tris–HCl (pH 7.5), 100 mm KCl, 5 mm MgCl2, 0.3% IGEPAL, 1X protease‐inhibitor cocktail (ThermoFisher Scientific), 100 U·mL^−1^ RNAse inhibitor (Thermo Fisher Scientific)] on ice for 10 min and centrifuged at 18 000 ***g*** for 10 min at 4°C to remove cell debris. 10% of the lysate was saved as input, whereas the remaining was incubated with the respective Streptavidin magnetic beads (Sigma) for biotinylated‐miRNA pulldown. 50 μL of Streptavidin magnetic beads per 10‐cm plate were washed twice with Solution A [0.1 m NaOH (Sigma), 0.05 m NaCl (ThermoFisher Scientific)], twice with Solution B [0.1 m NaCl (ThermoFisher Scientific)] and three times with Lysis buffer. The beads were then incubated with 10 μL of 10 mg·mL^−1^ yeast tRNA (Sigma) in lysis buffer at 4°C for 2 h. Blocked beads were washed once with lysis buffer and incubated with the cell lysates overnight at 4°C followed by total RNA isolation using phenol‐chloroform extraction and ethanol precipitation. Input RNA and pulldown RNA were used for cDNA synthesis and qRT‐PCR for miR‐30e and its related direct targets.

### 2.8 Xenograft tumorigenicity assay

Xenograft studies were performed using 6–8‐week‐old NSG (NOD.CgPrkdcscidIl2rgtm1Wjl/SzJ (005557) male mice (Jackson Laboratory, West Grove, PA, USA) maintained under pathogen‐free conditions. All xenograft experiments were performed according to relevant guidelines and regulations using an animal protocol approved by the Animal Ethics Committee/Institutional Animal Care and Use Committee of the University of Central Florida (IACUC #: 17–07). 22Rv‐1‐30e cells (6 × 10^6^ cells per mouse) were injected subcutaneously into the flank region in a total volume of 100 μL in a mixture with 0.1% matrigel (Corning, Corning, NY, USA). Once tumor volume crossed 150 mm^3^, animals were randomly divided into the uninduced group (4 mice per group) receiving regular feed and induced groups (4 mice per group) receiving doxycycline‐feed (625 mg doxycycline/kg, Envigo Teklad Diet, Boyertown, PA, USA)). Tumor growth was monitored weekly using a caliper and tumor volume was calculated as 0.52 × length × height × width. Once the animals reached the euthanization tumor volume (1500 mm^3^), the tumors were harvested and used for RNA extraction to check for expression of miR‐30e and specific mRNA targets by qRT‐PCR. Furthermore, tumor tissue architecture was monitored by H&E staining of FFPE sections.

### 2.9 Multiplex immunofluorescence staining

5 μm FFPE tissue sections were deparaffinized, rehydrated and incubated for 13 min in 1xTE in a Cusinart pressure cooker. After cooling down for 20 min, slides were incubated with primary antibodies (AR, cleaved PARP, and Ki67 rabbit antibodies: Cell Signaling Technology; Cytokeratin 8 antibodies: Santa Cruz) overnight at room temperature in 1xPBS/0.5% bovine serum albumin (BSA). Slides were washed with 1xPBS and incubated for 15 min with goat anti‐rabbit IgG horseradish peroxidase (HRP)‐conjugated (Vector Laboratories, Lowellville, OH, USA). Slides were incubated next with fluorescent dye‐conjugated tyramide from Biotium, i.e., FITC (0.5 mg/200 μL): 1 : 800 or CF555 (0.5 mg/200 μL): 1 : 200. Dilutions were made in a 0.1 m borate buffer with hydrogen peroxide added immediately before staining to a final concentration of 0.003%. Slides were incubated for 20 min at room temperature with the tyramide and then washed in 1xPBS. For the next round of staining, the antibodies were removed by heating in a microwave in 1xTE buffer. Then the second primary antibody was added for overnight incubation. For a 4‐antibody stain, the sequence started with FITC‐tyramide, followed by CF555‐tyramide. The last two antibodies were detected with a mix of anti‐rabbit IgG (H + L), F(ab’)2 Fragment Alexa Fluor® 647 Conjugate (Cell Signaling Technology) and Horse Anti‐Mouse Biotinylated Antibodies (H + L) (Vector Laboratories) at 1 : 400 dilution. After 15 min, slides were washed with 1xPBS and incubated with a Streptavidin‐Pacific Orange conjugate (ThermoFisher; 1 : 200) for 15 min. After final washes, slides were mounted with DAPI Fluoromount‐G (Southern Biotech, Birmingham, AL, USA) and imaged using a Leica SP5 confocal microscope (Leica Biosystems, Buffalo Grove, IL, USA). The antibody reactivity was calculated as the sum of percent of positivity and intensity of the stain (weak‐1, moderate‐2 or strong‐3).

### 2.10 RNA samples preparation for next‐generation RNA sequencing

Total RNA was extracted from 22Rv‐1‐30e stable lines with and without doxycycline induction and used for RNA Sequencing performed by LC Sciences. First, RNA integrity was checked using Agilent Technologies 2100 Bioanalyzer (Agilant Technologies, Santa Clara, CA, USA). Ribosomal RNA was depleted, and the sequencing library was prepared using Illumina's TruSeq‐stranded‐total‐RNA‐sample preparation protocol (Illumina, San Diego, CA, USA). This includes RNA fragmentation, reverse transcription using random primers, dUTP incorporation, A‐ tailing, adapter ligation, strand degradation and PCR enrichment. Illumina's NovaSeq 6000 sequencing (Illumina) was used to perform paired‐end sequencing. RNA quality control and library preparation were performed by LC Sciences (Houston, TX, USA).

### 2.11 RNA sequencing and data analysis

Reads that had adaptor contamination, low quality and undetermined bases were removed using Cutadapt and perl scripts, and sequence quality was validated using FastQC. All samples had a Q ≥ 30 score of > 98. The reads were mapped to the human genome (Version: v101) using bowtie2 [33] and hisat2 [34] and were assembled using stringtie [35]. All transcriptomes from six samples were merged to reconstruct a comprehensive transcriptome using perl scripts and gffcompare. StringTie was also used to calculate the expression levels of mRNAs and LncRNAs by using FPKM values, and the differentially expressed mRNAs were chosen based on log_2_ (fold change) > 1 or log_2_ (fold change) < −1 and with parametric *F*‐test comparing nested linear models (*P* value < 0.05) by r package edger [36]. For identification of lncRNA CPC [37] and CNCI [38] were utilized to predict transcripts with coding potential. The remaining transcripts with class codes (I, j, o, u, x) were considered as lncRNAs. The definition of class codes includes, j) Potentially novel isoform (fragment): at least one splice junction is shared with a reference transcript, i) A transfrag falling entirely within a reference intron, o) Generic exonic overlap with a reference transcript, u) Unknown, intergenic transcript and x) Exonic overlap with reference on the opposite strand. All transcripts with CPC score < 0.5 and CNCI score < 0 were removed. The remaining transcripts with class code were considered as lncRNAs. GO enrichment analysis was used to identify the differentially expressed genes enriched in specific GO terms, and those GO terms with *P* value ≤ 0.05 were defined to be significant. KEGG pathway analysis identified the enrichment of top pathways by the differentially expressed genes, and those with a *P* value of ≤ 0.05 were defined as significant KEGG pathways. Statistical computing and all other analysis were performed by LC Sciences. The sequencing coverage and quality statistics for each sample are summarized in Table **S1**.

### 2.12 Statistical analysis

All statistical analyses were performed using Student's *t*‐test or one‐way ANOVA for independent measures and Kaplan–Meier estimation for the longevity analysis using graphpad prizm (GraphPad Prizm, San Diego, CA, USA). We used a multinomial regression. The statistical significance was set at *P* < 0.05. Data were represented as mean ± SD.

## 3 Results

### 3.1 miR‐30e is downregulated in PCa cells and plays a role in regulating cell cycle, apoptosis and drug sensitivity of PCa cells

**Fig. 1:**
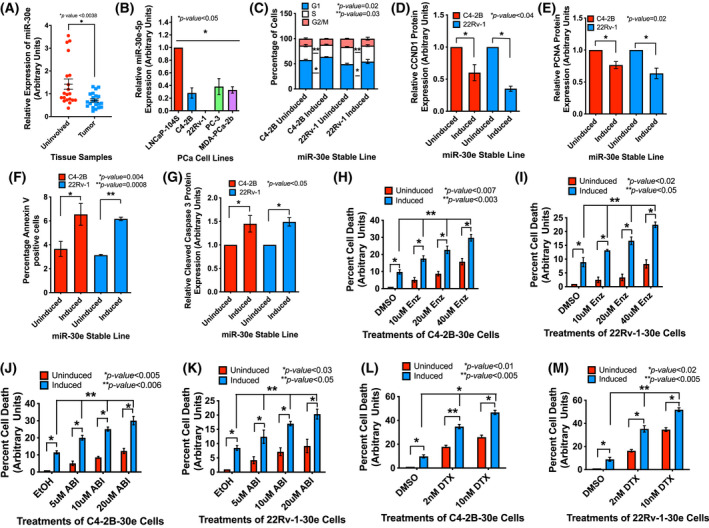
miR‐30e is downregulated in prostate tumors and changes in its expression regulates cell cycle progression, apoptosis and drug sensitivity. (A) Average fold change in expression of miR‐30e showing loss of expression in tumor tissues compared to matched normal tissues (*n* = 21). Data show the mean ± standard error of the mean (SEM). (B) Relative fold change in expression of miR‐30e by quantitative real‐time PCR (qRT‐PCR) showing decreased expression in all prostate cancer (PCa) cell lines with the least expression in the 22Rv‐1 cells. Raw data were normalized to the geomeans of U1, U6 and RNU43 small nuclear RNAs (snRNAs). Data show the mean ± standard deviation (SD) of three biological replicates. (C) Cell cycle analysis performed after 72 h of induction revealed significant increase in G1 population and decrease in the S phase cells in the induced C4‐2B‐30e and 22Rv‐1‐30e cells compared to the respective uninduced controls. Data show the mean ± SD of three biological replicates. (D) Densitometry analysis of G1 phase marker CCND1 showing decreased expression in the induced C4‐2B and 22Rv‐1 cells compared to the respective uninduced controls. Data show the mean ± SD of three biological replicates. (E) Densitometry analysis of S phase marker PCNA showing decreased expression in the induced C4‐2B‐30e and 22Rv‐1‐30e cells expressing miR‐30e compared to the respective uninduced controls. Results show the mean ± SD of three biological replicates. (F) Quantification of the apoptosis assay 24 h post induction exhibiting increased percentage of annexin V‐positive cells in the induced C4‐2B‐30e and 22Rv‐1‐30e cells compared to the respective uninduced controls. Results show the mean ± SD of three biological replicates. (G) Densitometry analysis of the pro‐apoptotic marker‐cleaved caspase3 showing increased expression in the induced C4‐2B‐30e and 22Rv‐1‐30e cells compared to the respective uninduced controls. Results show the mean ± SD of three biological replicates. (H, I) MTS assays showing a dose‐dependent increase in the percentage cell death of induced C4‐2B‐30e and 22Rv‐1‐30e cells to enzalutamide (ENZ) compared to uninduced controls and DMSO (vehicle control). Results show the mean ± SD of three biological replicates. (J, K) MTS assays showing dose‐dependent increase in percentage cell death in induced C4‐2B‐miR‐30e and 22Rv‐1‐miR‐30e cells treated with different doses of abiraterone (ABI) compared to uninduced controls and ethanol (vehicle control). Data show the mean ± SD of three biological replicates. (L, M) MTS assays showing dose‐dependent increase in percentage cell death in induced C4‐2B‐30e and 22Rv‐1‐30e cells treated with different doses of docetaxel (DTX) compared to uninduced controls and DMSO (vehicle control). Results show the mean ± SD of three biological replicates. All statistical analyses were performed using Student's *t*‐test in graphpad prizm with statistical significance set at *P < 0.05. [Colour figure can be viewed at wileyonlinelibrary.com]

We conducted microRNA expression profiling using PCa clinical samples. Selected patients had no treatments prior to surgical therapy, PSA‐before‐surgery ranged from 3.4 to 87.8 and Gleason Scores from 6 to 9. FFPE tissues (obtained from CHTN, NCI) were macro‐dissected for tumor and uninvolved areas and used for RNA extraction and qRT‐PCR. Based on the log_2_ fold change (FC) values, loss of expression of miR‐30e was noted in prostate tumors compared to uninvolved areas (Fig. 1A). Patient criteria have been mentioned in our published study [39]. We used the same RNA samples for this study. Differential expression of miR‐30e was also noted in PCa cells with various attributes with the lowest expression in aggressive CRPC cells 22Rv‐1 which express the AR‐V7 variant (Fig. 1B).

Functional analysis of miR‐30e on cell phenotype was conducted using three CRPC cell lines C4‐2B and 22Rv‐1 cells that express AR, and highly aggressive PC‐3 cells that do not express AR. We used ectopic expression approach and generated stable sublines C4‐2B‐30e, 22Rv‐1‐30e and PC‐3‐30e that showed 6‐10‐fold overexpression of miR‐30e upon induction with doxycycline, which is within the physiological range of miR‐30e expression (Fig. **S1**A). Analysis of cell cycle progression showed an increased population of G1 phase cells (~ 5%) and decreased population of S phase (~ 3–5%) in the induced C4‐2B‐30e and 22Rv‐1‐30e cells compared to the uninduced controls (Fig. 1C and Fig. **S1**B,C). The observation of sluggish G1 and S phase progression was complemented by the western blot data showing a significant reduction in expression of G1 and S phase markers, CCND1 (40% and 65%) and PCNA (24% and 37%), respectively, in C4‐2B‐30e and 22Rv‐1‐30e cells upon induction and also in induced PC‐3 cells (34% of CCND1 and 14% of PCNA) compared to uninduced cells (Fig. 1D,E and Fig. **S1**D,E). The results showing reduced expression of G1 and S phase markers upon expression of miR‐30e in PC‐3 cells are in support of the observation by Zheng *et al*. [40] showing a G1 cell cycle arrest in miR‐30e expressing PC‐3 cells (Fig. **S1**D–F).

As part of its tumor suppressor function, we studied the onset of apoptosis in cells expressing miR‐30e by Annexin V staining. Our results showed an increase in the Annexin V‐positive cells 24 h after induction with doxycycline in C4‐2B‐30e cells and 22Rv‐1‐30e cells (1.7–2‐fold increase) compared to the uninduced controls (Fig. 1F, Fig. **S1**G,H). Western blot analysis of the apoptotic marker, cleaved caspase 3, supported the Annexin V results showing increased cleaved caspase 3 expression upon miR‐30e overexpression in induced C4‐2B‐30e (1.4‐fold) and 22Rv‐1‐30e (1.5‐fold) compared to the uninduced controls (Fig. 1G and Fig. **S1**I).

To determine the benefit of the pro‐apoptotic function of miR‐30e on the sensitivity of the drugs used as the standard of care, we tested the effect of Enzalutamide (ENZ) and Abiraterone (ABI) as the wild‐type C4‐2B and 22Rv‐1 CRPC cells show poor sensitivity to ENZ and ABI, specifically 22Rv‐1 cells which constitutively express AR‐V7 variant. C4‐2B‐30e and 22RV‐1‐30e cells with or without induction with doxycycline were treated ENZ and ABI at different doses for 48 h. Our results showed a progressive and significant increase in cell death with increasing concentration of ENZ and ABI treatments of induced C4‐2B cells (Fig.1H,J). An average of 2.0‐, 2.3‐ and 3.0‐fold increase in cell death upon 10, 20 and 40 μm of ENZ treatments, respectively, and 2.0‐, 2.2‐, and 3.0‐fold increase in cell death upon 5, 10 and 20 μm of ABI treatments, respectively, compared to DMSO or ethanol, were noted in induced C4‐2B‐30e cells. 22Rv‐1‐30e cells also showed increased sensitivity albeit small, to ENZ and ABI upon induction with doxycycline. An average of 1.4‐, 2.0‐ and 2.3‐fold increased cell death upon ENZ treatments, respectively, and 1.5‐, 2.1‐ and 2.5‐fold increased cell death upon ABI treatments, respectively, compared to DMSO or ethanol, were noted in induced 22Rv‐1 cells (Fig.1I,K). Additionally, an average of 3‐ to 4‐fold increase in cell death in ENZ treated, and a 3‐fold increase in cell death in ABI treated C4‐2B and 22Rv‐1 cells were noted upon induction with doxycyclin compared to uninduced cells. Expression of miR‐30e in C4‐2B‐30e and 22Rv‐1 itself showed an average of 10‐fold increase in cell death.

Taken together, this study showed an additive effect of miR‐30e expression on the cell‐killing effects of ENZ and ABI treatments in AR‐positive CRPC PCa cells. The effect of miR‐30e expression on improving drug sensitivity of PCa cells with or without expression of a functional AR was further supported by our observation on treatments of a chemotherapeutic agent Docetaxel (DTX) in a dose‐dependent manner. C4‐2B‐30e, 22Rv‐1‐30e and PC‐3‐30e cells were treated with DTX for 72 h at different doses. Our results showed 3.5‐ and 4.7‐fold increased cell death upon DTX (2 and 10 nm) treatments in induced C4‐2B‐30e cells, 4.0‐ and 6.0‐fold increased cell death in induced 22Rv‐1‐30e cells and 2.0‐ to 3.0‐fold increased cell death in induced PC‐3‐30e cells compared to DMSO treated induced cells (Fig.1L,M and Fig. **S1**J). An average of 19‐fold increase in cell death upon expression of miR‐30e in the highly metastatic PC‐3 cells compared to uninduced cells were noted, which suggests the benefit of miR‐30e expression in AR‐negative CRPC PCa cells.

### 3.2 miR‐30e interacts with AR, FBXO45, SRSF7 and MYBL2 mRNAs and alters their expression in PCa cells

**Fig. 2:**
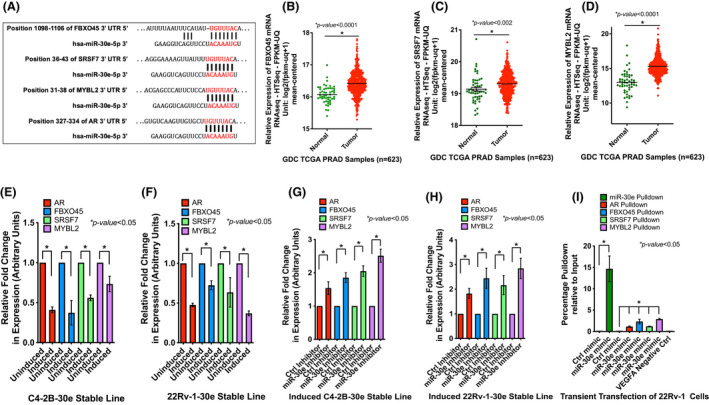
miR‐30e downregulated expression of its targets, AR, FBXO45, SRSF7 and MYBL2 mRNAs, that are upregulated in prostate tumors, and show direct interaction in the RISC. (A) *In silico* analysis revealed AR, FBXO45, SRSF7 and MYBL2 are potential targets of miR‐30e with predicted binding sites of miR‐30e in the 3’UTR of the target genes. (B, C and D) Expression analysis of the FBXO45, SRSF7 and MYBL2 mRNAs using The Cancer Genome Atlas (TCGA) Prostate Adenocarcinoma (PRAD) dataset (*n* = 623) showing significant upregulation of expression of FBXO45 (B), SRSF7 (C) and MYBL2 (D) in prostate tumors compared to the normal tissues. (E, F) Quantitative real‐time PCR (qRT‐PCR) analysis of the average fold change in expression of target mRNAs in C4‐2B‐30e and 22Rv‐1‐30e cells showing decreased expression of AR, FBXO45, SRSF7 and MYBL2 mRNAs in the induced C4‐2B‐30e (E) and 22Rv‐1‐30e (F) cells compared to uninduced controls. Data show the mean ± standard deviation (SD) of three independent analyses. (G, H) qRT‐PCR analysis of expression of the target mRNAs upon knockdown of miR‐30e in the induced C4‐2B‐30e and 22Rv‐1‐30e transfected with miR‐30e inhibitor or control inhibitor. Average fold change in expression revealed increased expression of AR, FBXO45, SRSF7 and MYBL2 mRNAs in the miR‐30e inhibitor transfected C4‐2B‐30e (G) and 22Rv‐1‐30e (H) cells compared to the control inhibitor transfected cells. Data show the mean ± SD of three independent analyses. (I) RNA immunoprecipitation (RNAIP) of 22Rv‐1 cells transfected with biotinylated miR‐30e mimic or control mimic. qRT‐PCR analysis showing pulldown of miR‐30e and the target mRNAs AR, FBXO45, SRSF7 and MYBL2 only in the biotinylated miR‐30e mimic‐transfected cells and not in the control mimic‐transfected cells. VEGFA, a not a target of miR‐30e, was used as an additional negative control which was not pulled down in either the miR‐30e mimic or control mimic‐transfected cells. Results show the mean ± SD of three independent analyses. All statistical analyses were performed using Student's *t*‐test in graphpad prizm with statistical significance set at *P < 0.05. [Colour figure can be viewed at wileyonlinelibrary.com]

To determine the function of miR‐30e in CRPC cells, we conducted target identification database search and filtered our search through TargetScan, miRDB and miRTar databases. Our analysis revealed AR, FBXO45, SRSF7 and MYBL2 mRNAs to be the top direct targets of miR‐30e (Fig. 2A). Among these targets, AR has already been established as the dominant target for therapy in aggressive CRPC. We examined the expression status of FBXO45 [41, 42], SRSF7 [43, 44] and MYBL2 [45], which are not well studied in PCa, in clinical specimens using the Cancer Genome Atlas (TCGA) database. UCSC Xena analysis of the TCGA PRAD dataset (*n* = 623) showed significant upregulation of FBXO45, SRSF7 and MYBL2 mRNAs in the PCa tumor tissues 50–60% compared to normal (Fig. 2B–D). To determine the relationship between miR‐30e and these target mRNAs, we tested the expression of these four mRNAs in CRPC cells upon expression of miR‐30e. qRT‐PCR analysis showed significant decrease in mRNA levels of AR (53–60%), FBXO45 (28–63%) SRSF7 (37–44%) and MYBL2 (63–27%) in induced 22Rv‐1‐30e and C4‐2B‐30e cells, respectively, compared to uninduced cells (Fig. 2E,F). Significant decrease in the steady‐state levels of mRNAs of FBXO45 (60%), SRSF7 (43%) and MYBL2 (49%) was also observed upon miR‐30e overexpression in the induced PC‐3‐30e cells compared to uninduced control (Fig. S2A).

The effect of miR‐30e on AR, FBXO45, SRSF7 and MYBL2 mRNAs was further confirmed by a knockdown approach to study the rescue effect using the miR‐30e (miR‐30e‐5p antagomirs) inhibitor. Transfection of the inhibitor showed > 90% inhibition of miR‐30e in both C4‐2B‐30e and 22Rv‐1‐30e induced cells compared to the control inhibitor (Fig. S2B). Induced C4‐2B‐30e and 22Rv‐1‐30e cells transfected with miR‐30e inhibitor showed a rescue effect with increased expression of AR (1.5‐ and 1.8‐fold), FBXO45 (1.8‐ and 2.4‐fold), SRSF7 (2.0 and 2.1‐fold) and MYBL2 (2.5‐ and 2.8‐fold) mRNAs, respectively, compared to the control inhibitor (Fig. 2G,H).

One of the main reasons of ADT failure in PCa is the appearance of the AR splice variant, AR‐V7 variant, which does not have a ligand‐binding domain [46]. We chose 22RV‐1 as our experimental model as these cells express both the full‐length AR and the AR‐V7 variant. As AR is one of the targets of miR‐30e, we examined if miR‐30e expression reduces both full‐length AR and the spice variant of AR. Western blot analysis showed significant downregulation of the full‐length AR (43% and 49%) in C4‐2B‐30e and 22RV‐1‐30e cells, respectively, and the AR‐V7 variant (44%) in 22Rv‐1‐30e cells upon induction compared to the cells without induction (Fig. S2C–E).

To examine the interaction of miR‐30e with its target mRNAs, we conducted RNA immunoprecipitation (RNAIP) from wild‐type 22RV‐1 cells using biotinylated miR‐30e mimic. qRT‐PCR analysis of the pulled‐down RNA complex showed the presence of miR‐30e and the target mRNAs (AR, FBXO45, SRSF7 and MYBL2), whereas the control mimic did not show precipitation of any of the target mRNAs. The RNAIP with miR‐30e mimic did not pull down VEGFA mRNA which is not targeted by miR‐30e, thereby confirming the specificity of miRNA and target mRNA interactions. RNAIP experiments substantiate miR‐30e and target mRNAs RISC complex formation (Fig. 2I). qRT‐PCR of the 10% input samples showed overexpression of miR‐30e and downregulation of the target mRNAs in the 22Rv‐1 cells transfected with biotinylated miR‐30e mimic compared to control mimic (Fig. S2F,G).

### 3.3 Expression of miR‐30e delayed tumor growth in xenograft models.

**Fig. 3:**
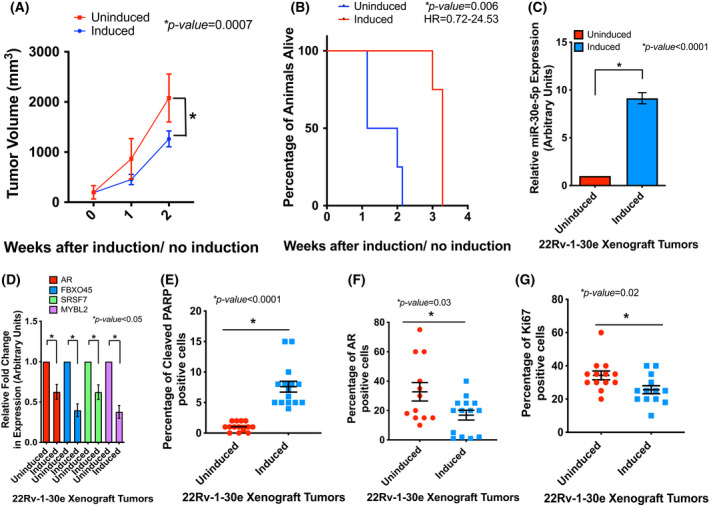
miR‐30e expression reduced tumor growth in xenograft mice. (A) 22Rv‐1‐30e xenograft tumor growth progression in mice with or without doxycycline induction with dox‐feed. Tumor volume measurement showed an overall slower tumor growth in the induced group compared to uninduced control group. Data show mean ± standard deviation (SD) of tumor volumes of 4 mice per group. Statistical analysis was performed using one‐way ANOVA with statistical significance set at *P < 0.05. (B) Mice with 22Rv‐1 tumors in dox‐feed group survived significantly longer based on the time to reach the specified euthanization cutoff tumor volume of 1.5 cm^3^. HR = Hazard ratio. Statistical analysis was performed using Kaplan–Meier estimation for the longevity analysis using GraphPad Prizm with multinomial regression with significance set at *P < 0.05 (*n* = 4). (C) Quantitative real‐time PCR (qRT‐PCR) analysis of the tumor tissues showing increased expression of miR‐30e in the induced group compared to the uninduced group. Data show mean ± SD of 4 tumors per group. Statistical analyses were performed using Student's *t*‐test in GraphPad Prizm with statistical significance set at *P < 0.05. (D) qRT‐PCR analysis of the mRNA targets of miR‐30e in the xenograft tumor tissues (AR, FBXO45, SRSF7 and MYBL2) showing significant decrease in expression in the doxycycline‐induced group compared to the uninduced control group. Data show mean ± SD of 4 tumors per group. Statistical analyses were performed using Student's *t*‐test in graphpad prizm with statistical significance set at *P < 0.05. (E–G) Multiplex tyramide staining for the pro‐apoptotic marker‐cleaved PARP, AR and proliferation marker Ki67 in the xenograft tumor tissues. Fluorescent signal from each marker was normalized to the fluorescent signal from cytokeratin 8. Data show mean ± standard error of the mean (SEM) of the fluorescent signals from randomly selected fields from two sections from each of the tumors per group (*n* = 3 tumors per group). Each data point represents a field with a percentage of cells with positive signals. Statistical analyses were performed using Student's *t*‐test in graphpad prizm with statistical significance set at *P < 0.05. Quantification of the fluorescent intensity revealed increased expression of cleaved PARP in induced tumors (15 fields) compared to uninduced tumors (14 fields) (E), decreased expression of AR in induced tumors (14 fields) compared to uninduced tumors (12 fields) (F) and decreased expression of Ki67 upon miR‐30e overexpression in the induced group (13 fields) compared to the uninduced control (13 fields) (G). [Colour figure can be viewed at wileyonlinelibrary.com]

Our *in vitro* studies so far revealed the antiproliferative role of miR‐30e and identified its multiple oncogenic targets in CRPC PCa cell lines. These observations prompted us to study the *in vivo* effect of miR‐30e using xenograft models. We used the inducible 22Rv‐1‐30e cells for examining tumor growth in mice. We monitored tumor growth with or without doxycycline (Dox) induction for 21 days since 22Rv‐1 cells formed very aggressive tumors within a shorter period of time. We observed that animals in the uninduced group had a higher tumor volume at 2 weeks compared to the animals in the induced group indicating that the animals receiving Dox had significantly slower growing tumors (*P* = 0.0007) that took longer to reach the 1.5 cm^3^ euthanization volume (Fig. 3A). We also noted that animals receiving doxycycline showed significantly longer survival to reach the euthanization volume (*P* = 0.006) compared to the uninduced control group (Fig. 3B).

Expression analysis of miR‐30e in the tumor tissues showed a significantly higher expression (an average of 9‐fold) in the induced group compared to the uninduced group (Fig. 3C). Expression of miR‐30e target mRNAs showed significant downregulation (AR 38%, FBXO45 61%, SRSF7 38% and MYBL2 63%) in the doxycycline‐induced groups compared to the uninduced control group (Fig. 3D). We also analyzed expression of AR (one of the primary targets of CRPC treatments), cleaved PARP (apoptotic marker) and Ki67 (proliferation marker) by immunohistochemistry (IHC) in paraffin‐embedded tumor tissues. Our analysis showed a significant increase in cleaved PARP and a significant decrease in AR and Ki67 expression in all the animals from the induced group receiving doxycycline compared to the uninduced controls (Fig. 3E–G). Fluorescent intensities in all images were normalized to the expression of Cytokeratin 8, and H&E staining was performed to visualize tumor tissue architecture (Fig. S3). Overall, this observation confirms the antitumor role of miR‐30e *in vivo* through direct and indirect modulation of specific genes involved in apoptosis and cell cycle that was observed in PCa cell lines.

### 3.4 Transcriptome analysis showed altered gene expression in PCa cells expressing miR‐30e

To understand the tumor suppressor effect of miR‐30e on global gene expression in PCa cells, we performed RNA‐seq analysis of the doxycycline‐induced (group E) and ‐uninduced 22Rv‐1‐30e (group C) cells. The reads were aligned to the Human Genome (Version: v101) using Bowtie2 [33] and HISAT2 [34]. Sequencing statistics of each sample is presented in Table **S1**.

**Fig. 4:**
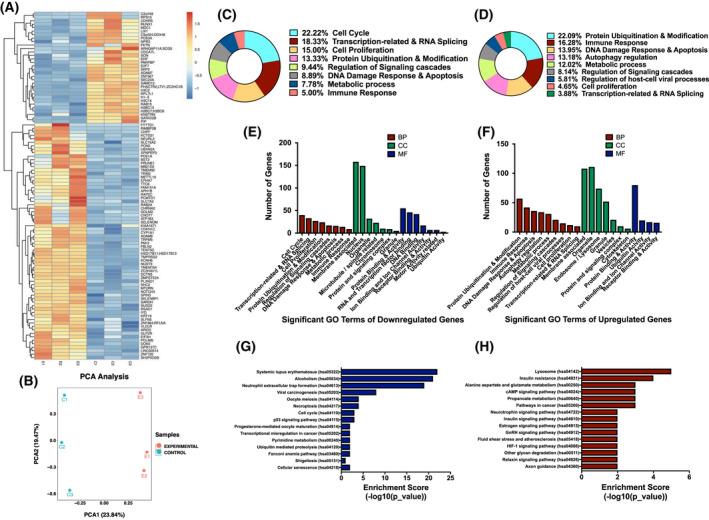
Transcriptome profiling revealed a set of genes that are significantly dysregulated miR‐30e expressing 22Rv‐1 cells. (A) Hierarchical clustering analysis showing top differentially expressed genes between the induced 22Rv‐1‐30e cells (E group) compared to uninduced control group (C group). The rows represent the genes, whereas the columns represent the samples. Red color indicates higher expression and blue color indicates lower expression. Statistical analysis using parametric *F*‐test comparing nested linear models with *P < 0.05 significance by r package edger. (B). Principle component analysis (PCA) of three biological replicates of induced 22Rv‐1 cells (E group) and uninduced group (C group) showing distinct differences in gene expression profiles between two groups. (C, D) Gene Ontology (GO) enrichment analysis for the differentially expressed genes using the Fisher exact *t*‐test assuming binomial distribution in Enrichr software with *P ≤ 0.05 showing the circular plots of top altered GO terms based on biological processes (BP) of downregulated (C) and upregulated genes (D). (E) Bar chart showing the number of genes altered for specific GO term for downregulated genes based on 8 biological processes (BP), 7 cellular components (CC) and 7 molecular functions (MF). (F) Bar chart showing the number of genes altered for specific GO term for upregulated genes based on 8 biological processes (BP), 7 cellular components (CC) and 7 molecular functions (MF). (G) KEGG pathway analysis for the downregulated genes using the Fisher exact *t*‐test assuming binomial distribution in Enrichr software with *P ≤ 0.05 showing the bar chart depicting the top 15 pathways that were altered for the downregulated genes in the induced 22Rv‐1‐30e cells compared to the uninduced control. (H) KEGG pathway analysis for the upregulated genes using the Fisher exact *t*‐test assuming binomial distribution in Enrichr software with *P ≤ 0.05 showing the bar chart depicting the top 15 pathways that were altered for the downregulated genes in the induced 22Rv‐1‐30e cells compared to the uninduced control. [Colour figure can be viewed at wileyonlinelibrary.com]

The FPKM (Fragments Per Kilobase of transcript per Million) values showing the abundance of the differentially expressed genes and transcripts between induced and uninduced groups were estimated by the StringTie [35]. Pearson correlation analysis showed a strong correlation (> 0.988) of genes between induced and uninduced 22Rv‐1‐30e cells. (Fig. S4A). Using R package edgeR [36], 138 upregulated and 97 downregulated genes were selected with log_2_ (fold change) > 1 or log_2_ (fold change) < −1 and with parametric *F*‐test comparing nested linear models (*P* value < 0.05). A volcano plot was created showing the differential expression of genes (Fig. S4B and Table S2). Based on the cutoff of *P* < 0.05 and fold change > 1.5, 431 upregulated and 315 downregulated genes were identified in miR‐30e expressing cells compared to the uninduced control cells Table S3. Unsupervised hierarchical clustering based on FPKM values of differentially expressed genes showed distinct groups of genes that are upregulated and downregulated in experimental (E induced) and control group of cells (C uninduced) (Fig. 4A and Table S4). Principal component analysis (PCA) shows distinct grouping of samples with genes that have *P* value < 0.05 on FPKM abundance estimation (Fig. 4B). We also noted expression of a large number of long noncoding RNA in these group of cells. Analysis of the relative expression levels based on log_10_ FPKM values showed a comparable pool of lncRNA expression in these group of cells (Fig. S4C,D). Taken together, transcriptome analysis revealed alteration of expression of a distinct set of genes upon expression of miR‐30e in 22Rv‐1 PCa cells.

### 3.5 Identification of differentially expressed genes that are functionally related, and enrichment of specific signaling pathways upon expression of miR‐30e

Gene Ontology (GO) analysis was performed based on specific gene attributes that include biological processes (BP), cellular components (CC) and molecular function (MF). Briefly, all upregulated and downregulated genes with *P* value ≤ 0.05 were filtered and inputted into Enrichr, a comprehensive gene set enrichment analysis tool [47], which classified the genes into specific groups under BP, CC and MF. The top 100 differentially expressed genes were used to construct the circular plots for BP, CC and MF to show the significantly enriched GO terms for downregulated (Fig. 4C, Fig. S5A,B, and Tables S5–S7) and upregulated genes (Fig. 4D, Fig. S5C,D and Tables S8–S10). Furthermore, bar graphs of the significantly enriched GO terms for BP, CC and MF showed the number of upregulated and downregulated genes for each term (Fig. 4E,F). The top BP GO terms that were enriched for the differentially expressed genes belonged to cell cycle progression, protein ubiquitination, apoptosis and transcription‐related processes that support our observations from the *in vitro* and *in vivo* studies on miR‐30e tumor suppressor role in PCa.

KEGG Pathway analysis of upregulated and downregulated genes revealed multiple pathways that were significantly enriched (Fig. 4G (downregulated) and 4H (upregulated) and Figs S6 and S7). The top 15 KEGG pathways of downregulated genes and upregulated genes were plotted as a bar graph (Fig. 4G,H and Tables S11 and S12). Some of the top altered pathways for the downregulated genes involved ubiquitin‐mediated proteolytic pathway (Fig. S6A), cell cycle (Fig. S6B), necroptosis (Fig. S6C) and p53 signaling (Fig. S6D) pathway. For the upregulated genes, some of the top altered pathways include metabolic pathway (Fig. S7A), lysosome (Fig. S7B) and pathways in cancer (Fig. S7C). Overall, GO and KEGG pathway analysis established that miR‐30e expression is closely involved in regulating expression of genes involved in various biological processes, molecular functions and signaling pathways that could have a significant impact in PCa progression.

### 3.6 miRNA‐30e‐modulated expression of genes involved in cell cycle, apoptosis and ubiquitination that may induce tumor suppressor effects of miR‐30e

**Fig. 5:**
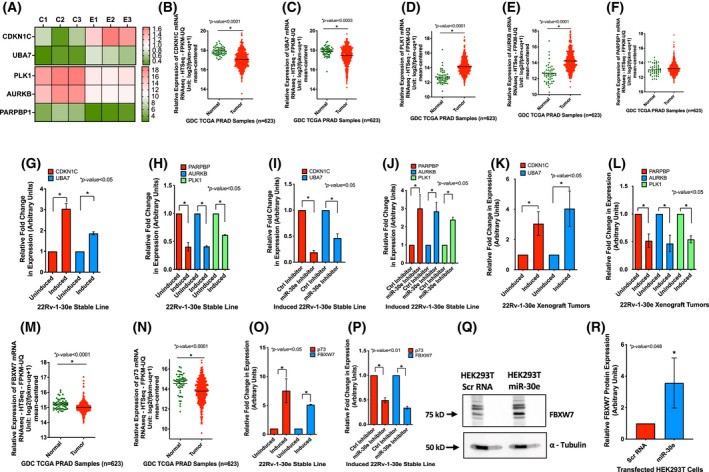
Functional analysis and validation of the dysregulated genes in miR‐30e expressing cells. (A) Expression heatmap showing altered expression of genes involved in cell cycle (CDKN1C, PLK1 and AURKB), apoptosis (PARPBP1) and ubiquitination (UBA7) in miR‐30e expressing 22Rv‐1 cells (E group) compared to uninduced control cells (C group). Values from three biological replicates are shown with statistical analysis using parametric *F*‐test comparing nested linear models with *P < 0.05 significance by r package edger. (B–F) Analysis of The Cancer Genome Atlas (TCGA) Prostate Adenocarcinoma (PRAD) dataset (*n* = 623) showing significant downregulation of CDKN1C and UBA7 (B, C) and upregulation of PLK1, AURKB and PARPBP1 (D–F) expression in prostate cancer (PCa) tumors compared to normal tissues. Statistical analyses were performed using Student's *t*‐test in graphpad prizm with statistical significance set at *P < 0.05. (G, H) Validation of expression of the selected genes by quantitative real‐time PCR (qRT‐PCR) showing significantly increased expression of CDKN1C and UBA7 (G) and significantly decreased expression of PARPBP, AURKB and PLK1 (H) upon overexpression of miR‐30e in 22Rv‐1‐30e cells compared to uninduced control cells. Data show the mean ± standard deviation (SD) of three independent analyses with statistical analyses performed using Student's *t*‐test in graphpad prizm with statistical significance set at *P < 0.05. (I, J) confirmation of the rescue effect of knockdown of miR‐30e on expression of selected genes in the induced 22Rv‐1‐30e cells transfected with miR‐30e inhibitor or control inhibitor. qRT‐PCR analysis showed significant decrease in expression of CDKN1C and UBA7 (I) and significant increase in expression of PARPBP, AURKB and PLK1 (J) in miR‐30e inhibitor treated cells. Data show the mean ± SD of three independent analyses. Statistical analyses performed using Student's *t*‐test in graphpad prizm with statistical significance set at *P < 0.05. (K, L) qRT‐PCR analysis of expression of the selected genes in 22Rv‐1‐30e xenograft tumors showing significant upregulation of CDKN1C and UBA7 *expression* (K) and significant downregulation of PARPBP, AURKB and PLK1 expression (L) upon overexpression of miR‐30e in the induced mice group compared to uninduced control mice group. Results show the mean ± SD of three independent analyses. Statistical analyses performed using Student's *t*‐test in graphpad prizm with statistical significance set at *P < 0.05. (M, N) miR‐30e regulates ubiquitination through stabilization of FBXO45 downstream target FBXW7 and p73. Analysis of TCGA PRAD dataset (*n* = 623) showing significant downregulation of FBXW7 (M) and p73 (N) in PCa tumors compared to normal tissues. Statistical analyses performed using Student's *t*‐test in GraphPad Prizm with statistical significance set at *P < 0.05. (O) qRT‐PCR analysis of FBXW7 and p73 expression showing upregulation of both mRNAs upon induction of miR‐30e expression in 22Rv‐1 cells compared to uninduced control cells. Data show the mean ± SD of three independent analyses. Statistical analyses performed using Student's *t*‐test in graphpad prizm with statistical significance set at *P < 0.05. (P) Inhibition of miR‐30 expression in induced 22Rv‐1‐30e cells using miR‐30 inhibitor showed decreased expression of FBXW7 and p73 in these cells compared to control cells. Data show the mean ± SD of three independent analyses. Statistical analyses performed using Student's *t*‐test in graphpad prizm with statistical significance set at *P < 0.05. (Q, R) western blot analysis of HEK293T cells expressing miR‐30e showing increased FBXW7 in miR‐30e vector transfected cells compared to control vector transfected cells. A representative image showing FBXW7 and other isoforms (Q) and densitometric analysis (R) of the normalized intensity of the FBXW7 band. Alpha‐tubulin was used as the loading control. Data show the mean ± SD of two separate experiments. Statistical analyses performed using Student's *t*‐test in graphpad prizm with statistical significance set at *P < 0.05. [Colour figure can be viewed at wileyonlinelibrary.com]

Our *in vitro* and *in vivo* analyses showed altered expression of miR‐30e target mRNAs, translation products of which are involved in cell cycle progression, apoptosis and ubiquitination. Our top GO enrichment term analysis also showed alteration of all three biological processes. These findings prompted us to analyze the RNA‐seq data further which revealed a set of dysregulated (≥ 1.5 fold) genes/mRNAs in induced 22Rv‐1‐30e cells compared to uninduced control cells that specifically involved in cell cycle (CDKN1C, AURKB, PLK1), apoptosis (PARPBP1) and ubiquitination (UBA7) (Fig. 5A) (Table S13). We next determined the clinical significance of expression of these genes in PCa progression through TCGA PRAD dataset (*n* = 623) analysis. We noted downregulation of CDKN1C (p57^kip2^) [48] and UBA7 [49] (Fig. 5B,C) and upregulation of PLK1 [50], AurkB [51] and PARPBP1 [52] (Fig. 5D–F) mRNAs in PCa tumors compared to the normal prostate epithelium. qRT‐PCR analysis showed significant overexpression of mRNAs of two upregulated genes CDKN1C and UBA7 and reduced expression of three downregulated genes PARPBP1, AURKB and PLK1 in the induced 22Rv‐1‐30e (Fig. 5G,H) and C4‐2B‐30e (Fig. S8A,B) cells compared to the uninduced control cells. Similar expression patterns of these mRNAs were noted in induced 22Rv‐1‐30e xenograft tumors compared to the uninduced tumors (Fig. 5K,L). Inhibition of miR‐30e expression by miR‐30e antagomirs reversed the expression patterns of the upregulated and downregulated mRNAs in induced 22Rv‐1‐30e (Fig. 5I,J) and C4‐2B‐30e (Fig. S8C,D) cells.

Our *in vitro*, *in vivo* and transcriptome analyses upon overexpression of miR‐30e showed dysregulation of two key genes. FBXO45 (downregulated expression) and UBA7 (upregulated expression) involved in ubiquitination. To understand the effect of miR‐30e on modulation of ubiquitin proteosome pathway (UPS), we examined expression of FBXW7, a E3 ubiquitin ligase tumor suppressor and one of the targets of FBXO45 [53]. We also examined expression of p73 mRNAs, which is also a target for ubiquitination [42]. We first determined the mRNA expression status of FBXW7 and p73 in PCa tumors to understand their clinical significance as tumor suppressors using TCGA PRAD dataset (*n* = 623) analysis, which confirmed their loss of expression in PCa tumors compared to normal prostate tissues. (Fig. 5M,N). We also noted significant upregulation of FBXW7 and p73 mRNAs in induced 22Rv‐1‐30e (Fig. 5O) and C4‐2B‐30e (Fig. S8E) cells compared to uninduced control cells. We also noted increased expression of FBXW7 and p73 mRNA levels in doxycycline‐induced 22Rv‐1‐30e xenograft tumors compared to uninduced tumors (Fig. S8F).

To confirm the effect of miR‐30e, we used miR‐30e antagomirs to inhibit miR‐30e expression, which showed significant reduction in expression of FBXW7 and p73 mRNAs in induced 22Rv‐1‐30e (Fig. 5P) and C4‐2B‐30e (Fig. S8G) cells. Western blot analysis of HEK393T cells upon transient transfection of inducible miR‐30e expressing vector or control vector revealed a significant increase in FBXW7 expression (3.6‐fold) in miR‐30e expressing cells compared to control cells (Fig. 5Q,R). However, we did not observe any stabilization of p73 in induced HEK293T upon expression of miR‐30e (data not shown). It can be speculated that this effect could be mediated partly by miR‐30e‐induced degradation of FBXO45 mRNA leading to a possible reduction in the protein level, and thereby stabilization of FBXW7 and partly by stabilization of mRNAs through an indirect effect of miR‐30e as a result of its tumor suppressor function.

We also evaluated expression of KLK3 (PSA) as a transcriptional target of AR in PCa tumors since miR‐30e directly targets AR and regulates its expression. Analysis of TCGA PRAD dataset showed a significant increase in expression of KLK3 in PCa tissues compared to normal samples (Fig. S8H). We also noted a reduced expression of KLK3 upon overexpression of miR‐30e‐5p in both induced 22Rv‐1‐30e and C4‐2B‐30e cells compared to the uninduced controls (Fig. S8I). Antagomir‐mediated knockdown of miR‐30e expression in induced 22Rv‐1‐miR‐30e and C4‐2B‐miR‐30e cells significantly increased the expression of KLK3 (Fig. S8J). Altogether, our findings demonstrate that miR‐30e‐5p regulates a group of genes involved in cell cycle, apoptosis and ubiquitination.

### 3.7 miRNA‐30e displayed a reciprocal relationship with *HELLPAR* noncoding macroRNA in PCa cells

**Fig. 6:**
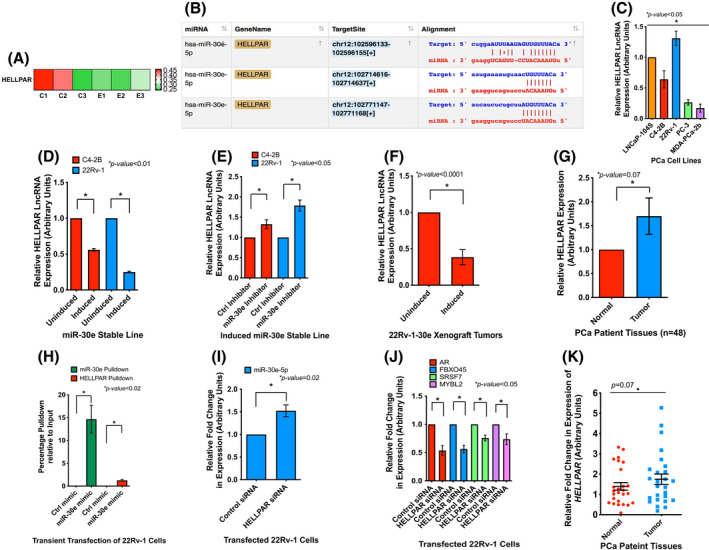
miR‐30e regulates and is regulated by LncRNA *HELLPAR* (a). Expression heatmap showing altered expression of *HELLPAR* long noncoding RNA (lncRNA) in induced 22Rv‐1‐30e cells compared to uninduced control cells. Values from three biological replicates are shown with statistical analysis using parametric *F*‐test comparing nested linear models with *P < 0.05 significance by r package edger. (B) *In silico* analysis of ENCORI database revealed 3 predicted binding sites of miR‐30e on *HELLPAR*. (C). Quantitative real‐time PCR (qRT‐PCR) analysis of the endogenous expression of *HELLPAR* in different prostate cancer (PCa) cells showing a maximum expression in 22Rv‐1 cells compared to other cells. Raw data was normalized to the expression of TBP housekeeping gene. Data show the mean ± standard deviation (SD) of three separate experiments. Statistical analyses performed using Student's *t*‐test in graphpad prizm with statistical significance set at *P < 0.05. (D) qRT‐PCR analysis of miR‐30e expressing induced C4‐2B‐miR‐30e and induced 22Rv‐1‐miR‐30e cells showing decreased expression of *HELLPAR* compared to the uninduced controls. Data show the mean ± SD of three separate experiments. Statistical analyses performed using Student's *t*‐test in graphpad prizm with statistical significance set at *P < 0.05. (E) qRT‐PCR analysis of miR‐30e knockdown in the induced C4‐2B‐miR‐30e and induced 22Rv‐1‐miR‐30e cells showing increased expression of *HELLPAR* compared to the control inhibitor transfected cells. Data show the mean ± SD of three separate experiments. Statistical analyses performed using Student's *t*‐test in graphpad prism with statistical significance set at *P < 0.05. (F) qRT‐PCR analysis of miR‐30e expressing induced xenograft tumor tissues showing decreased expression of *HELLPAR* compared to the uninduced xenograft group (*n* = 4). Statistical analyses performed using Student's *t*‐test in graphpad prizm with statistical significance set at *P < 0.05. (G) qRT‐PCR analysis of *HELLPAR* in PCa patient tissues (*n* = 48) showing an increased expression in tumor samples compared to matched normal samples. Statistical analyses performed using Student's *t*‐test in graphpad prizm with statistical significance set at *P = 0.07. (H) RNA immunoprecipitation of 22Rv‐1 cells transfected with biotinylated miR‐30e mimic or control mimic. qRT‐PCR analysis revealed pulldown of miR‐30e and *HELLPAR* LncRNA only in the biotinylated miR‐30e mimic‐transfected cells and not in the control mimic‐transfected cells. Data show the mean ± SD of three separate experiments. Statistical analyses performed using Student's *t*‐test in graphpad prizm with statistical significance set at *P < 0.05. (I) Knockdown of *HELLPAR* in wild‐type 22Rv‐1 cells followed by qRT‐PCR analysis showing increased expression of miR‐30e in the *HELLPAR*‐siRNA‐transfected 22Rv‐1 cells compared to control siRNA transfected cells. Data show the mean ± SD of three separate experiments. Statistical analyses performed using Student's *t*‐test in graphpad prizm with statistical significance set at *P < 0.05. (J) Knockdown of *HELLPAR* in wild‐type 22Rv‐1 cells followed by qRT‐PCR analysis showing decreased expression of the target mRNAs of miR‐30e (AR, FBXO45, SRSF7 and MYBL2) in the *HELLPAR*‐siRNA‐transfected 22Rv‐1 cells compared to control siRNA transfected cells. Data show the mean ± SD of at least 3 independent experiments. Statistical analyses performed using Student's *t*‐test in graphpad prizm with statistical significance set at *P < 0.05. (K) qRT‐PCR scatter plot analysis of *HELLPAR* in PCa patient tissues (*n* = 48) showing an increased expression in tumor samples compared to matched normal samples. Data show the mean ± standard error of the mean (SEM) of 48 samples. Statistical analyses performed using Student's *t*‐test in graphpad prizm with statistical significance set at *P = 0.07. [Colour figure can be viewed at wileyonlinelibrary.com]

As miRNAs are often sequestered by other noncoding RNAs (ncRNA), we were interested in learning about any other ncRNA, specifically lncRNAs, that might interact with miR‐30e. Analysis of the RNA‐seq data on lncRNA‐mRNA target prediction showed a possible relationship between *HELLPAR* macroRNA and PARPB1, one of the genes that showed downregulation upon miR‐30e expression as described above. *HELLPAR* is a novel lncRNA expressed as a single large transcript (205 012 bases) involved in the HELLP pregnancy‐associated syndrome [28]. Characterization of *HELLPAR* revealed its strong involvement with cell cycle progression [28] and its interaction with ribosomal and RNA‐splicing proteins [54]. These studies established a role of *HELLPAR* on regulating genes related to cell cycle although its significance in cancer has not been completely understood. Our transcriptome profiling identified *HELLPAR* as one of the lncRNAs which showed a decreased expression in miR‐30e expressing 22Rv‐1 stable line compared to the uninduced control (Fig. 6A). The use of ENCORI [55] prediction tool for lncRNA and mRNA interaction showed a trend of a positive correlation of expression between *HELLPAR* and PARPBP (Fig. S9A) and three potential binding sites of miR‐30e on *HELLPAR* RNA sequence (Fig. 6B). *HELLPAR* lncRNA showed differential expression in PCa cells of various characteristics with highest expression in 22Rv‐1 cells that express the AR‐V7 variant. (Fig. 6C and Fig. S9B).

The effect of miR‐30e on *HELLPAR* expression was examined next. qRT‐PCR using two different sets of primers showed that overexpression of miR‐30e led to a significant downregulation of *HELLPAR* in 22Rv‐1‐30e (~ 0.25‐fold and ~ 0.17‐fold respectively) and C4‐2B‐30e (~ 0.56‐fold and ~ 0.39‐fold, respectively) cells compared to uninduced control cells, which validated our observation upon RNA‐seq data analysis (Fig. 6D and Fig. S9C). All amplification results using primer set #2 are shown in the supplementary materials. The effect of miR‐30e on *HELLPAR* was confirmed by a knockdown approach using an miR‐30e inhibitor. Knockdown of miR‐30e showed an increased expression of *HELLPAR* in the induced 22Rv‐1‐30e (1.8‐ and 1.7‐fold) and C4‐2B‐30e (1.3‐ and 1.6‐fold) cells compared to the induced cells transfected with the control inhibitor (Fig. 6E and Fig. S9D). Analysis of the xenograft tumor tissues also showed significant downregulation of *HELLPAR* in the doxycycline‐treated groups (~ 0.38‐fold and ~ 0.2‐fold) compared to the uninduced control groups (Fig. 6F and Fig. S9E). Expression analysis of *HELLPAR* RNA in prostate tumors (*n*_tumor_ = 24) and matching uninvolved tissues (*n*_normal_ = 24) by qRT‐PCR using primer set #1 showed an upregulation (1.7‐fold) of *HELLPAR* in tumor tissues compared to uninvolved areas (Fig. 6G,K). Patient criteria and TNM classification of the tumor stages of selected patients ranged from 1 to 3 (Table S14).

**Fig. 7:**
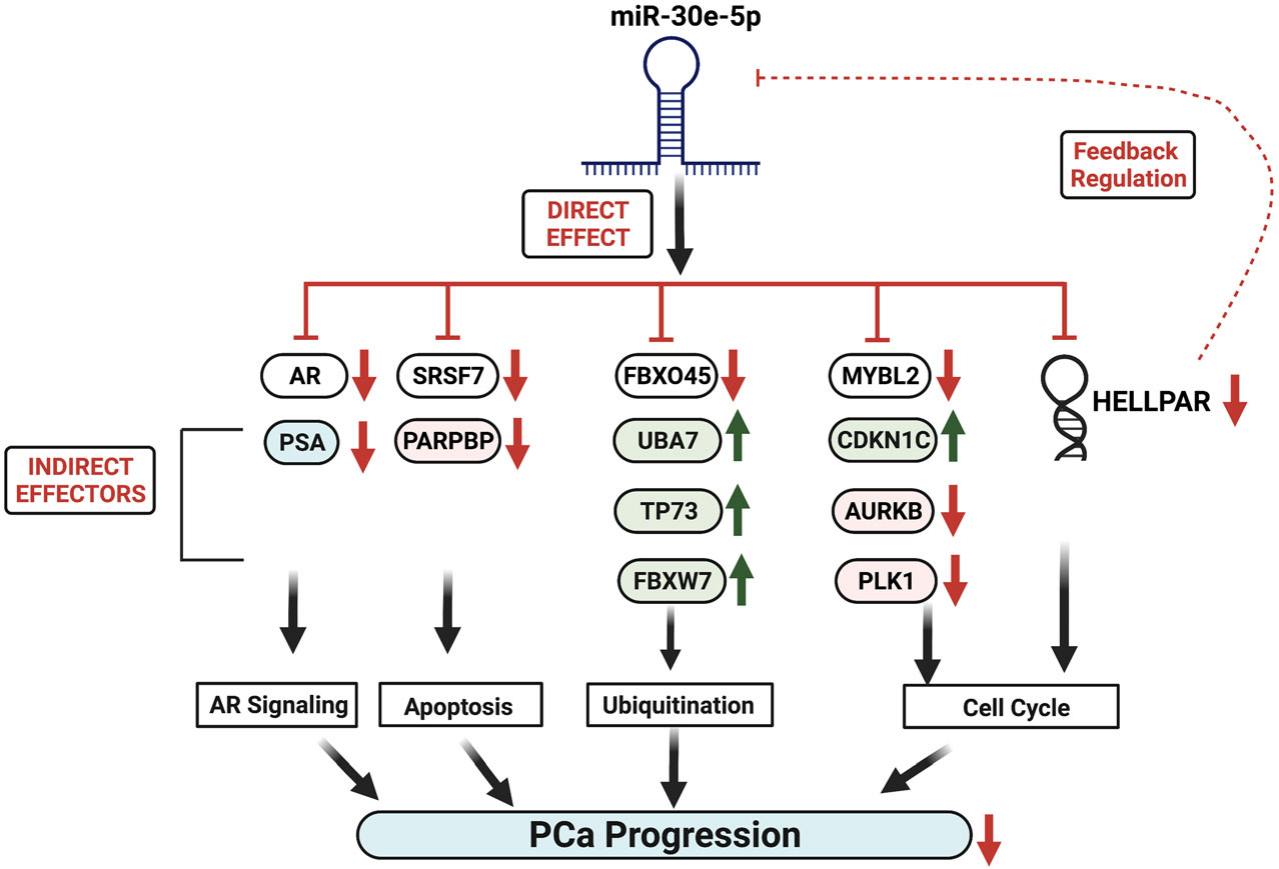
miR‐30e exerts its tumor suppressor role through modulation of miRNA:lncRNA:mRNA signaling axes. White boxes: Direct targets green boxes: Tumor suppressors; pink boxes: Oncogenes. Red arrows: Downregulation, green arrows: Upregulation. [Colour figure can be viewed at wileyonlinelibrary.com]

miR‐30e and *HELLPAR* interactions were examined by RNAIP using parental 22Rv‐1 cells and biotinylated miR‐30e mimic, which showed enrichment of *HELLPAR* in the immunoprecipitate of miR‐30e mimic compared to the control nonspecific RNA (Fig. 6H). qRT‐PCR analysis of the 10% input sample revealed the decreased expression of *HELLPAR* as expected, in the 22Rv‐1 cells transfected with miR‐30e mimic compared to the control mimic (Fig. S9F). These data confirmed a direct interaction between *HELLPAR* and miR‐30e. To test if *HELLPAR* has any reciprocal relationship with miR‐30e expression, we performed knockdown of *HELLPAR* in 22Rv‐1 cells transfected with *HELLPAR*‐siRNA. We obtained 60% decrease in *HELLPAR* expression in the *HELLPAR*‐siRNA‐transfected cells compared to control cells (Fig. S9G,H). Inhibition of *HELLPAR* in 22RV‐1 cells also exhibited an increased (1.5‐fold) expression of miR‐30e and decreased expression of miR‐30e target mRNAs (AR 46% decrease; FBXO45 44% decrease; SRSF7 25% decrease; MYBL2 24% decrease) compared to control (Fig. 6I,J). These results confirmed the reciprocal relationship between *HELLPAR* and miR‐30e both *in vitro* and *in vivo* and may play a role in regulating PCa progression. Overall, our study establishes the involvement of specific miRNA:lncRNA:mRNA regulatory network for executing the tumor suppressor role of miR‐30e in PCa progression. (Fig. 7).

## 4 Discussion

In this study, we investigated the tumor suppressor function of miR‐30e through regulation of specific target mRNAs and a macroRNA that modulate multiple signaling pathways in PCa. miR‐30 family function as tumor suppressors in many cancers, including PCa [56]. Of these, miR‐30e or miR‐30e‐5p exerts its tumor suppressor function by targeting specific genes such as *IRS1* in breast cancer [57], *HOXA1* in lung carcinomas [58], *ITGA6* and *ITGB1* in colorectal cancer [59], *MTDH* (Metadherin) in bladder cancer [60] and USP22/Sirt1/JAK/STAT3 signaling pathway in nonsmall cell lung cancer [61]. miR‐30e also interacts with other lncRNAs to negatively regulate cancer progression such as oncogenic lncRNA DLEU2 that promoted esophageal cancer through the miR‐30e/E2F7 axis [62] and MALAT1 which acted as a molecular sponge of miR‐30e to regulate ATG5 expression and autophagy in gastric cancer [63]. An oncogenic role of miR‐30e also has been reported that showed miR‐30e played a dual role based on the status of TP53 through the miR‐30e‐3p/TP53/MDM2 signaling axis [64]. Specifically in PCa, miR‐30e exhibited a tumor suppressor role by targeting genes such as *CHRM3*, *CTHRC1* [65] and genes in MAPK signaling pathway [40].

Our study presented novel information on tumor suppressor role of miR‐30e by demonstrating loss of expression of miR‐30e in prostate tumors compared to uninvolved areas, and miR‐30e overexpression mediated G1 arrest with decreased S phase cells. Our study also showed a pro‐apoptotic role through increased expression of cleaved caspase 3 and a potential benefit of miR‐30e replenishment on improving drug sensitivity of both AR‐positive and AR‐negative PCa cells. Expression of miR‐30e led to increased sensitivity of C4‐2B and 22Rv‐1 cells to ENZ, ABI and DTX and PC‐3 cells to DTX. The antitumor effect of miR‐30e was further confirmed *in vivo* showing decreased tumor volume and tumor progression along with decreased expression of the proliferation marker Ki67, and increased expression of apoptotic marker‐cleaved PARP in our xenograft studies.

We identified AR, which has already been established as a target of other miR‐30 family members and three novel targets of miR‐30e, FBXO45, SRSF7 and MYBL2, that are involved in cell cycle, apoptosis and ubiquitination pathways. Specifically in PCa, miR‐30b and miR‐30d are shown to directly target AR to regulate AR signaling axis in CRPC [58], but our results are the first that showed a relationship of miR‐30e with AR and AR signaling in PCa. The novel targets of miR‐30e are known oncogenes in many cancers including PCa. FBXO45 is a E3 ubiquitin ligase that is overexpressed in many cancers, and high expression of FBXO45 is also correlated with poor survival in many cancers [41]. FBXO45 targets the pro‐apoptotic Par‐4, first identified in prostate cells for proteosomal degradation resulting in increased survival of cancer cells [66]. SRSF7 belongs to a family of Serine/Arginine‐rich splicing factors and is involved in the regulation of the apoptotic signaling cascade through alternative splicing of the Fas ligand [67]. SRSF7 is upregulated in colon and lung cancer, and its knockdown inhibited cell proliferation and increased apoptosis [68]. Furthermore, SRSF7 regulated p21‐dependent cell cycle arrest in colon cancer cells [69]. In PCa, SRSF7 has been identified to be a negative prognostic biomarker [70] and specifically overexpressed in African American PCa [71]. SRSF7 was also shown to regulate and is regulated by miR‐30a in renal cancer [72], but there is no information regarding the functional relationship between miR‐30e and SRSF7 in PCa available. MYBL2, a known oncogene in many cancers, functions as a master regulator of cell proliferation, apoptosis, cell cycle progression, drug resistance and metastasis [73]. In PCa, MYBL2 plays a key role in castration resistance and metastasis by targeting the Hippo‐YAP pathway [74]. All members of the miR‐30 family target MYBL2 in hepatocellular carcinoma [75] and inhibition of MYBL2 expression by miR‐30a in PCa [76] and nonsmall cell lung cancer [77] were reported earlier. Nonetheless, our studies are the first to demonstrate a functional relationship between miR‐30e and MYBL2 in PCa. We validated interactions of miR‐30e with its targets by RNAIP and regulation of their mRNA levels by miR‐30e through knock in and silencing of miR‐30e expression PCa cells. Our results on expression analysis miR‐30e targets in xenograft tumors supported our *in vitro* results showing significant reduction of all four mRNAs in tumors in the induced group of mice (Fig. 3D). Overall, our study shows the role of miR‐30e as a tumor suppressor in PCa through regulation of key target oncogenic mRNAs that are overexpressed in PCa and involved in dysregulation of cell cycle, apoptosis and ubiquitination signaling axes. This observation was further evaluated by unbiased RNA‐seq analysis with or without expression of miR‐30e.

Our transcriptome profiling studies further complement the phenotypic characterization of the effects of miR‐30e overexpression. RNA‐seq data provided a comprehensive understanding on genes and pathways that facilitate tumor suppressor roles of miR‐30e. GO enrichment and KEGG pathway revealed a set of dysregulated genes, CDKN1C, PLK1, AURKB, PARPBP1 and UBA7 that are involved in cell cycle, apoptosis and ubiquitination pathways. TCGA PRAD dataset analysis supported our RNA‐seq data showing a reciprocal expression pattern with upregulation of AURKB, PLK1 and PARPBP1 and downregulation of CDKNIC and UBA7 in PCa tumors, which were validated by qRT‐PCR. AURKB, an established oncogene, is overexpressed in many cancers including PCa [78], which drives tumorigenesis via cell proliferation, cell cycle progression and cell survival [79, 80]. AURKB selective inhibitor AZD2811 which is formulated as a nanoparticle is currently in clinical trials as monotherapy and in combination to treat acute myeloid leukemia [81]. PLK1, an established oncogene in many cancers, is frequently overexpressed in glioma, colorectal cancer, breast cancer and in head and neck cancer [82]. PLK1 is frequently overexpressed in PCa and is involved in tumorigenesis. PLK1 signaling is also one of the top signaling pathways activated in CRPC and drives PCa in an androgen‐independent manner [83, 84]. PARPBP, also known as PARP1 binding protein, plays an important role in inhibition of homologous recombination during DNA repair. PARPBP1 has a dysregulated expression pattern in lung, pancreatic, cervical and gastric cancers and is linked to poor clinical outcomes. Increased PARPBP1 expression drives breast cancer and is a marker of breast cancer prognosis and chemo resistance [85]. PARPBP1 expression is enhanced by AR variants in PCa in the absence of full‐length AR, and the variants, in turn, interact with and are regulated by PARP1/2 in CRPC [86].

CDKN1C, a CDK inhibitor belonging to the Cip/Kip family, regulates various cancer hallmarks such as cell proliferation, cell cycle progression and apoptosis [87]. CDKN1C is frequently downregulated and plays a tumor suppressor role in many cancers including liver cancer, pancreatic cancer, lung cancer and breast cancer [88]. In PCa, CDKN1C was shown to have a tumor suppressor role and was inhibited by the oncogenic miR‐21 [89]. UBA7, an E1‐like‐ubiquitin‐activating enzyme, plays a major role in the conjugation of ISG15 in a process known as ISGylation which regulate the activity of target proteins. UBA7/ISG15 signaling axis has been a prime focus in lung cancer, and many studies show downregulation of UBA7 in lung and breast cancer [49, 90]. ISGylation has been implicated in PCa progression by regulating AR expression although there is no information regarding the specific role of UBA7 in PCa progression [91].

Our observation on upregulated expression of the tumor suppressors E3 ligase FBXW7 and p73, as revealed by increased mRNA levels and stabilization of FBXW7 in immunoblot analysis upon miR‐30e overexpression, further supports the speculation that miR‐30e modulates genes involved in ubiquitin pathway. This is also identified in RNA‐seq analysis as one of the top biological process that was dysregulated upon miR‐30e overexpression. FBXW7, a member of the SCF E3 ligase family, functions as a tumor suppressor and contributes to CRPC [92]. We identified that FBXO45 mRNA is a target of miR‐30e. Recent studies showed that FBXO45, an E3 ubiquitin ligase and a member of the SCF E3 ligase family [41], regulates expression of FBXW7 through direct binding and ubiquitination [53]. FBXO45 also directly target and degrade p73 and knockdown of FBXO45 stabilized p73 and induced apoptosis in a p53‐independent manner [42].

Another interesting observation is the inverse association of miR‐30e with *HELLPAR* macroRNA [28], which is a target of miR‐30e and which directly interacts with the miRNA. No study is available that characterized the functional significance of *HELLPAR* in cancer progression although a previous study proposed a potential role of *HELLPAR* as a ceRNA of miR‐30d‐5p and a regulator of the miR‐30d‐GJA1 signaling axis in pancreatic cancer [93]. *HELLPAR* interacts with the key ribosomal and RNA‐splicing proteins that are upregulated in multiple cancers [54]. Our study provided convincing evidence on miR‐30e‐mediated modulation of expression of *HELLPAR* in PCa cells and in xenograft tumors. Interestingly, siRNA‐mediated reduction of *HELLPAR* expression showed increased expression of miR‐30e and decreased expression of its target mRNAs, AR, FBXO45, SRSF7 and MYBL2, which further confirm *HELLPAR*‐mediated regulation of the functional miR‐30e pool possibly acting as a ceRNA, and miR‐30e itself regulates *HELLPAR* expression. The reciprocal relationship suggests that both RNAs maintain a functional balance that depends on the aggressiveness of the prostate tumors. Further studies are required to fully understand the function of *HELLPAR* in PCa.

## 5 Conclusion

Overall, this study provides convincing evidence that miR‐30e exerts a tumor suppressor role through modulation of *HELLPAR* lncRNA and a number of key oncogenic mRNAs that are clinically significant in PCa and are involved in different signaling pathways, including AR signaling [94], cell cycle progression [95, 96], apoptosis [97] and ubiquitination pathways [98]. Thus, our study demonstrates that miR‐30e has a multidimensional role in PCa progression and indicates the significance of miR‐30e as a potential therapeutic target for PCa.

## Conflict of interest

The authors declare no conflict of interest.

## Author contributions

RC and KG designed the study. KG, CN, RO and TA contributed to carry out the experiments. KG, CN, RO and TA contributed to analyzing data. RC supervised the data analysis. JP contributed by providing patients' clinical information, experimental data and analyzing data of for *HELLPAR* LncRNA. DC analyzed the immunofluorescence staining of xenograft tumor tissues. KG and RC wrote the manuscript. RC and TA supervised the research. All authors read and approved the final manuscript.

## Supporting information


**Fig. S1.** Expression of miR‐30e in different PCa cells reduced cell cycle, induced apoptosis and improved the drug sensitivity of PC‐3 cells.
**Fig. S2.** Expression of miR‐30e reduced expression of FBXO45, SRSF7 and MYBL2 mRNAs in PC‐3‐30e cells and AR in C4‐2B‐30e and 22Rv‐1‐30e cells.
**Fig. S3.** Multiplex tyramide staining of miR‐30e expressing xenograft tumor tissues revealed altered expression of proliferation and apoptosis markers.
**Fig. S4.** RNA sequencing analysis revealed altered coding and noncoding gene expression in miR‐30e expressing cells compared to control.
**Fig. S5.** RNA sequencing analysis revealed altered Gene Ontology terms in miR‐30e expressing cells compared to control.
**Fig. S6.** miR‐30e expression revealed top KEGG pathways that were enriched for the downregulated genes.
**Fig. S7.** miR‐30e expression revealed top KEGG pathways that were enriched for the upregulated genes.
**Fig. S8.** miR‐30e overexpression revealed decreased expression of genes involved in cell cycle progression, apoptosis, ubiquitination and AR signaling.
**Fig. S9.** A reciprocal functional relationship between miR‐30e and HELLPAR.
**Table S1.** Sequencing statistics of each sample used for RNA‐seq.
**Table S2.** Differentially expressed genes_Upregulated and Downregulated (separate excel sheet).
**Table S3.** Volcano plot of all differentially expressed genes (separate excel sheet).
**Table S4.** Heatmap differentially expressed genes_upregulated and downregulated (separate excel sheet).
**Table S5.** BP downregulated genes (separate excel sheet).
**Table S6.** BP upregulated genes (separate excel sheet).
**Table S7.** CC downregulated genes (separate excel sheet).
**Table S8.** CC upregulated genes (separate excel sheet).
**Table S9.** MF downregulated genes (separate excel sheet).
**Table S10.** MF upregulated genes (separate excel sheet).
**Table S11.** KEGG downregulated genes (separate excel sheet).
**Table S12.** KEGG upregulated genes (separate excel sheet).
**Table S13.** Selected RNA‐seq genes for validation.
**Table S14.** Patient criteria for tissues used for HELLPAR expression.

## Data Availability

The RNA‐seq data generated in this study are available in GEO under accession number # GSE188345. All other data that support the findings of this study are available from the corresponding author (ratna.chakrabarti@ucf.edu) upon reasonable request.
