# Supplementary material for: Anticancer function of microRNA‐30e is mediated by negative regulation of HELLPAR , a noncoding macroRNA, and genes involved in ubiquitination and cell cycle progression in prostate cancer
Source: Mol Oncol. 2022 Jun 14;16(16):2936–58. doi: 10.1002/1878-0261.13255 (PMC9394257; doi:10.1002/1878-0261.13255)
Supplement: Supplementary file 1 — Fig. S1. Expression of miR‐30e in different PCa cells reduced cell cycle, induced apoptosis and improved the drug sensitivity of PC‐3 cells. Fig. S2. Expression of miR‐30e reduced expression of FBXO45, SRSF7 and MYBL2 mRNAs in PC‐3‐30e cells and AR in C4‐2B‐30e and 22Rv‐1‐30e cells. Fig. S3. Multiplex tyramide staining of miR‐30e expressing xenograft tumor tissues revealed altered expression of proliferation and apoptosis markers. Fig. S4. RNA sequencing analysis revealed altered coding and noncoding gene expression in miR‐30e expressing cells compared to control. Fig. S5. RNA sequencing analysis revealed altered Gene Ontology terms in miR‐30e expressing cells compared to control. Fig. S6. miR‐30e expression revealed top KEGG pathways that were enriched for the downregulated genes. Fig. S7. miR‐30e expression revealed top KEGG pathways that were enriched for the upregulated genes. Fig. S8. miR‐30e overexpression revealed decreased expression of genes involved in cell cycle progression, apoptosis, ubiquitination and AR signaling. Fig. S9. A reciprocal functional relationship between miR‐30e and HELLPAR. Table S1. Sequencing statistics of each sample used for RNA‐seq. Table S2. Differentially expressed genes_Upregulated and Downregulated (separate excel sheet). Table S3. Volcano plot of all differentially expressed genes (separate excel sheet). Table S4. Heatmap differentially expressed genes_upregulated and downregulated (separate excel sheet). Table S5. BP downregulated genes (separate excel sheet). Table S6. BP upregulated genes (separate excel sheet). Table S7. CC downregulated genes (separate excel sheet). Table S8. CC upregulated genes (separate excel sheet). Table S9. MF downregulated genes (separate excel sheet). Table S10. MF upregulated genes (separate excel sheet). Table S11. KEGG downregulated genes (separate excel sheet). Table S12. KEGG upregulated genes (separate excel sheet). Table S13. Selected RNA‐seq genes for validation. Table S14. Patient criteria [file MOL2-16-2936-s001.pdf]

Supplemental Information

Anti-cancer function of microRNA-30e is mediated by negative regulation of *HELLPAR,* a noncoding macroRNA, and genes involved in ubiquitination and cell cycle progression in prostate cancer

Kavya Ganapathy[1], Christopher Ngo[1], Thomas Andl[1], Domenico Coppola[3, 4], Jong Park[2] and Ratna Chakrabarti[1]*

Table of Contents: **Page Numbers**

**Supplementary Tables**	**2-4**
Supplementary Table 1	2
Supplementary Table 2	Available in separate file
Supplementary Table 3	Available in separate file
Supplementary Table 4	Available in separate file
Supplementary Table 5	Available in separate file
Supplementary Table 6	Available in separate file
Supplementary Table 7	Available in separate file
Supplementary Table 8	Available in separate file
Supplementary Table 9	Available in separate file
Supplementary Table 10	Available in separate file
Supplementary Table 11	Available in separate file
Supplementary Table 12	Available in separate file
Supplementary Table 13	3
Supplementary Table 14	4
**Supplementary Figures**	**5-13**
Figure S1	5
Figure S2	6
Figure S3	7
Figure S4	8
Figure S5	9
Figure S6	10
Figure S7	11
Figure S8	12
Figure S9	13

Supplementary Table 1

Table 1: Sequencing statistics of each sample used for RNA-Seq

Sample ID	Total number of sequenced reads	Total number of uniquely Mapped reads	RNA integrity number (RIN)	Ratio of all reads aligned to rRNA regions to total uniquely mapped reads (rRNA rate)	Ratio of exon-mapped reads to total uniquely mapped reads (Expression Profile Efficiency)	Total number of detected transcripts with reads ≥1b	Total number of detected transcripts with reads ≥1b
C1	67686044	32875052	9.2	< 0.01%	99.98%	5226	4807
C2	54644772	27189179	9.6	< 0.01%	99.98%	5102	
C3	51821414	19328983	8.6	< 0.01%	99.99%	5150	
E1	65578118	29954680	9	< 0.01%	99.98%	5252	4763
E2	65241524	25508148	9.6	< 0.01%	99.98%	5075	
E3	64835186	24934260	9.1	< 0.01%	99.99%	5106	

Table 13: Selected RNA-Seq Genes for Validation

GENES	EXPRESSION	PATHWAY
AURKB	1.5-fold downregulated	Cell cycle
PLK1	1.5-fold downregulated	Cell cycle
PARPBP	2-fold downregulated	Apoptosis
CDKN1C	2.7-fold upregulated	Cell cycle
UBA7	2.3-fold upregulated	Ubiquitination

Supplementary Table 14

Table 14. Patient criteria for tissues used for *HELLPAR* expression

Patient #	Stage	Age at Diagnosis	Race	Vital Status
1	2	53	White	Alive
2	2	67	White	Alive
3	2	54	White	Alive
4	2	50	White	Alive
5	2	61	Black	Alive
6	2	53	White	Alive
7	2	61	White	Alive
8	3	58	White	Alive
9	3	59	White	Dead
10	1	75	White	Dead
11	3	70	White	Dead
12	3	64	White	Dead
13	3	62	White	Alive
14	1	74	White	Dead
15	2	58	White	Alive
16	2	64	White	Dead
17	2	71	White	Dead
18	2	56	White	Alive
19	2	46	White	Alive
20	3	61	White	Dead
21	2	46	White	Alive
22	1	71	White	Dead
23	2	45	White	Alive
24	2	63	White	Dead

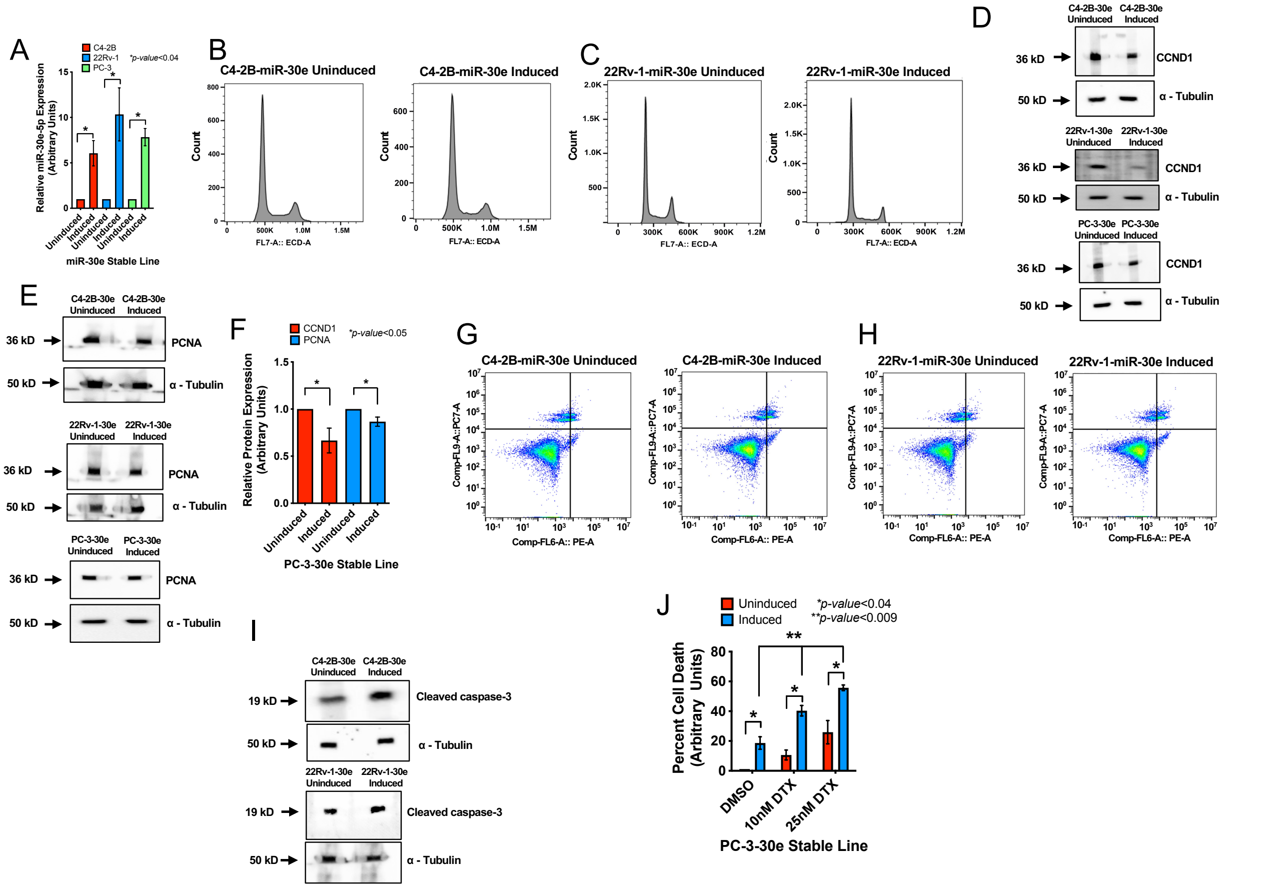

FIGURE S1

Fig. S1. Expression of miR-30e in different PCa cells reduced cell cycle, induced apoptosis and improved the drug sensitivity of PC-3 cells. (A) Fold change in miR-30e expression in the stable C4-2B, 22Rv and PC-3 PCa cells showing significant upregulation in induced cell lines compared to the uninduced controls. (**B, C)** Two parameter histogram showing cell cycle progression of miR-30e expressing induced C4-2B and 22Rv-1 cells compared to the respective uninduced controls. (**D)** Representative Western Blot images of CCND1 (G1 marker) and loading control α-tubulin in miR-30e expressing induced C4-2B, 22Rv-1 and PC-3 stable lines compared to respective uninduced controls. **(E)** Representative Western Blot images of PCNA (S-phase marker) and loading control α-tubulin in miR-30e expressing induced C4-2B, 22Rv-1 and PC-3 stable lines compared to respective uninduced controls. **(F)** Densitometry analysis of PC-3-30e cells western blots for CCND1 and PCNA revealed decreased expression of CCND1 and PCNA in the induced PC-3 cells expressing miR-30e compared to the uninduced control. **(G** and **H)** Flow cytometric analysis of Annexin V expression of the apoptosis assay for miR-30e expressing induced C4-2B and 22Rv-1 stable lines compared to the respective uninduced controls. **[I]** Representative Western Blot images of cleaved-caspase 3 (pro-apoptotic marker) and loading control α-tubulin in miR-30e expressing induced C4-2B and 22Rv-1 stable lines compared to respective uninduced controls. **(J)** PC-3-30e subline cells were induced or uninduced and treated with different doses of Docetaxel (10nM and 25nM). MTS assay revealed increased cell death in a dose-dependent manner in the induced PC-3 cells expressing miR-30e compared to the uninduced control and DMSO which was used as the vehicle control. All data show the mean ± SD of at least 3 independent experiments.

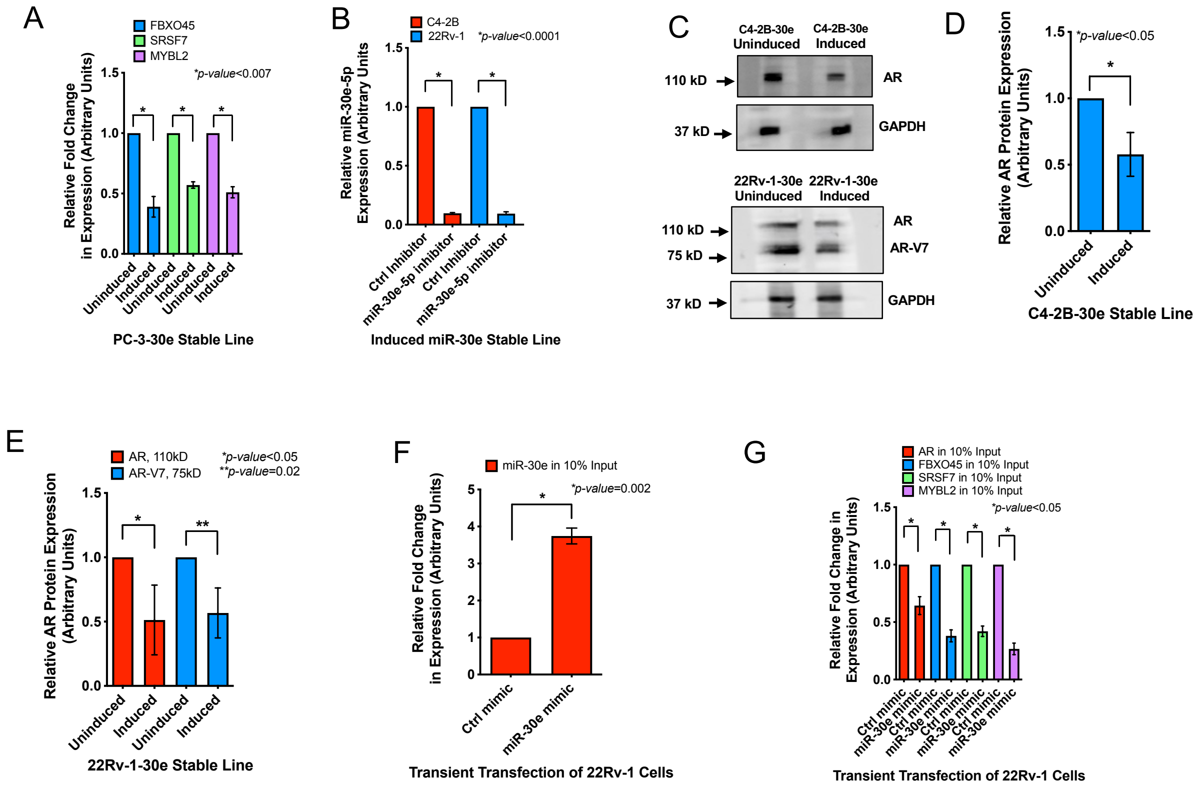

FIGURE S2

Fig. S2. Expression of miR-30e reduced expression of FBXO45, SRSF7 and MYBL2 mRNAs in PC-3-30e cells and AR in C4-2B-30e and 22Rv-1-30e cells. (A) qRT-PCR analysis revealed decreased expression of FBXO45, SRSF7 and MYBL2 mRNAs in the induced PC-3-30e expressing cells compared to the uninduced control. **(B)** Knockdown of miR-30e in the stable C4-2B-30e and 22Rv-1-30e cells confirmed decreased expression of miR-30e in the induced C4-2B-30e and 22Rv-1-30e cells transfected with miR-30e inhibitor compared to control inhibitor. **(C)** Representative Western Blot images of AR and loading control GAPDH in miR-30e expressing induced or uninduced C4-2B-30e and 22Rv-1-30e cells. **(D, E)** Densitometry analysis of the western blots revealed decreased expression of full length AR in the induced C4-2B-30e cells **(D)** compared to the uninduced control as well as a decreased expression in full-length AR and AR-v-7 in the induced 22Rv-1-30e cells (**E**) compared to the uninduced control. **(F)** RNA immunoprecipitation of 22Rv-1 cells transfected with biotinylated miR-30e mimic or control mimic. qRT-PCR analysis of the 10% input samples revealed increased expression of miR-30e in the 22Rv-1 cells transfected with the biotinylated miR-30e mimic compared to the control mimic. **(G)** RNA immunoprecipitation of 22Rv-1 cells transfected with biotinylated miR-30e mimic or control mimic. qRT-PCR analysis of the 10% input samples revealed decreased expression of the mRNA targets (AR, FBXO45, SRSF7 and MYBL2) of miR-30e in the 22Rv-1 cells transfected with the biotinylated miR-30e mimic compared to the control mimic. All data show the mean ± SD of at least 3 independent experiments.

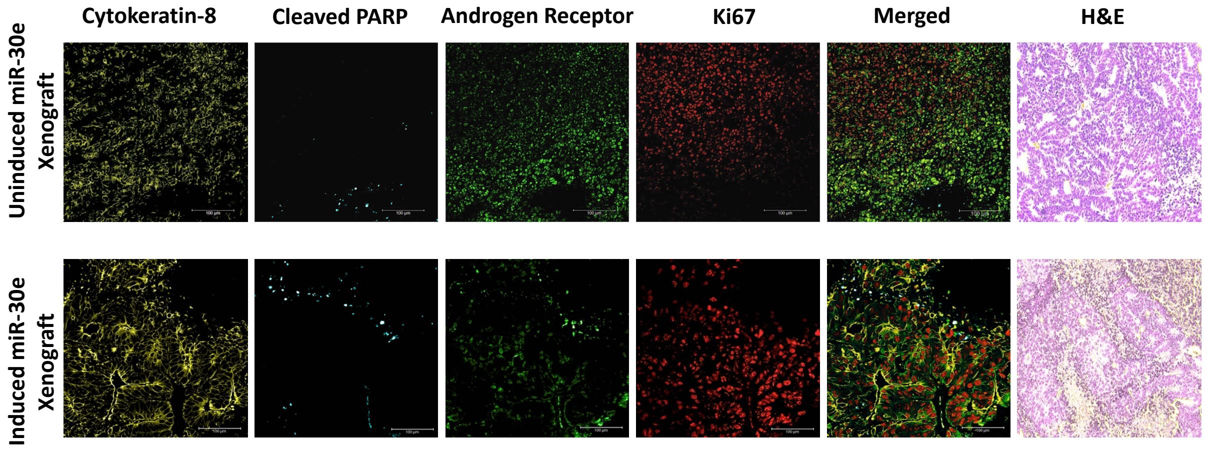

FIGURE S3

Fig. S3. Multiplex Tyramide staining of miR-30e expressing xenograft tumor tissues revealed altered expression of proliferation and apoptosis markers. Immunofluorescence analysis of cleaved PARP (apoptotic marker), AR (direct target of miR-30e) and Ki67 (proliferation marker) expression in mice tumor tissues from induced group expressing miR-30e (bottom panel) compared to the uninduced control group (top panel). Representative images showed a noticeable decrease in Ki67 expression (red) and AR expression (green) and increased expression in cleaved PARP (Cyan) in the induced tumors compared to the uninduced tumor tissues. Cytokeratin-8 (yellow) was used as the tumor cell marker. H&E-stained images show the histology of tumor tissues. Scale bar: 100 µm

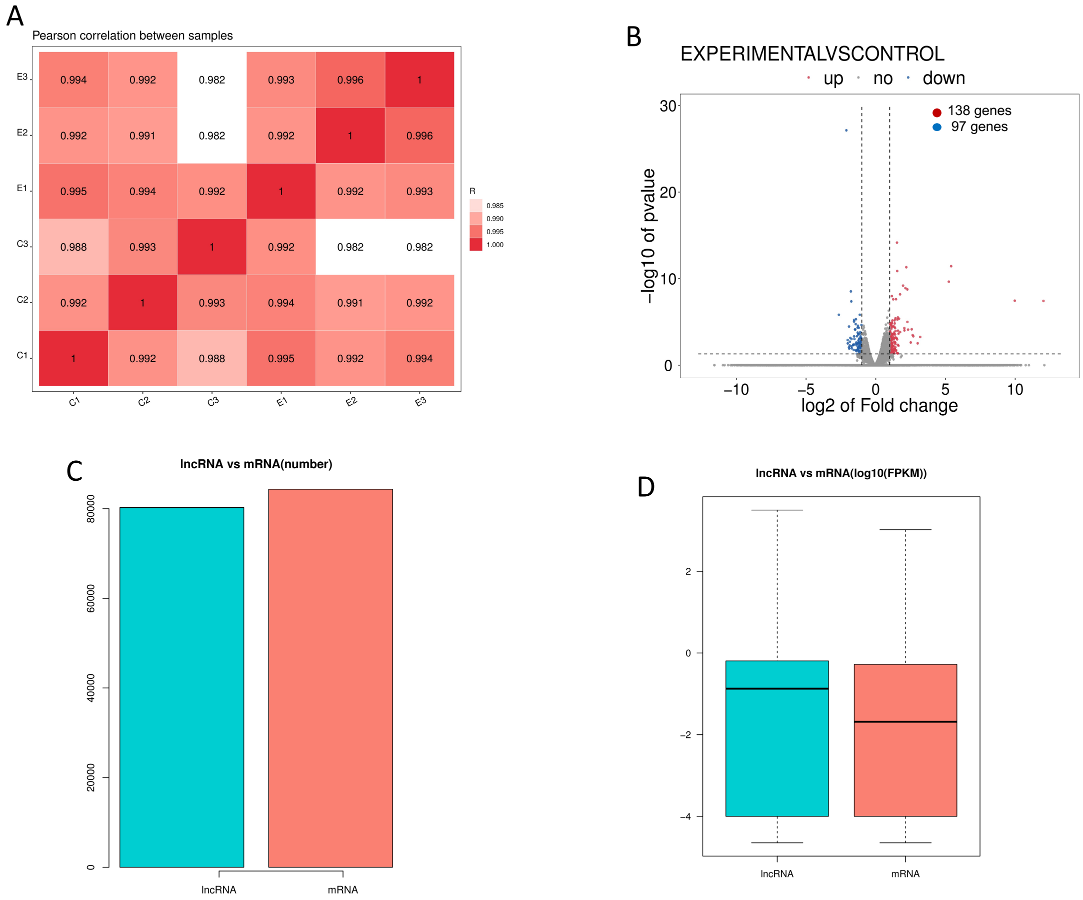

FIGURE S4

Fig. S4. RNA sequencing analysis revealed altered coding and noncoding gene expression in miR-30e expressing cells compared to control. (**A**) Pearson correlation of gene level expression of all samples showing the relations among samples above 0.980. (**B**). Volcano plot revealed a large magnitude of dysregulated genes between the induced 22Rv-1-30e cells compared to uninduced controls. The $\log_2$ fold change in gene expression was plotted on the X-axis with the $\log_{10}$ of the p-values plotted on the Y-axis. (**C, D**). Comparative analysis of the relationship between number of expressed lncRNAs and mRNAs (**C**) and their respective average FPKM values (**D**).

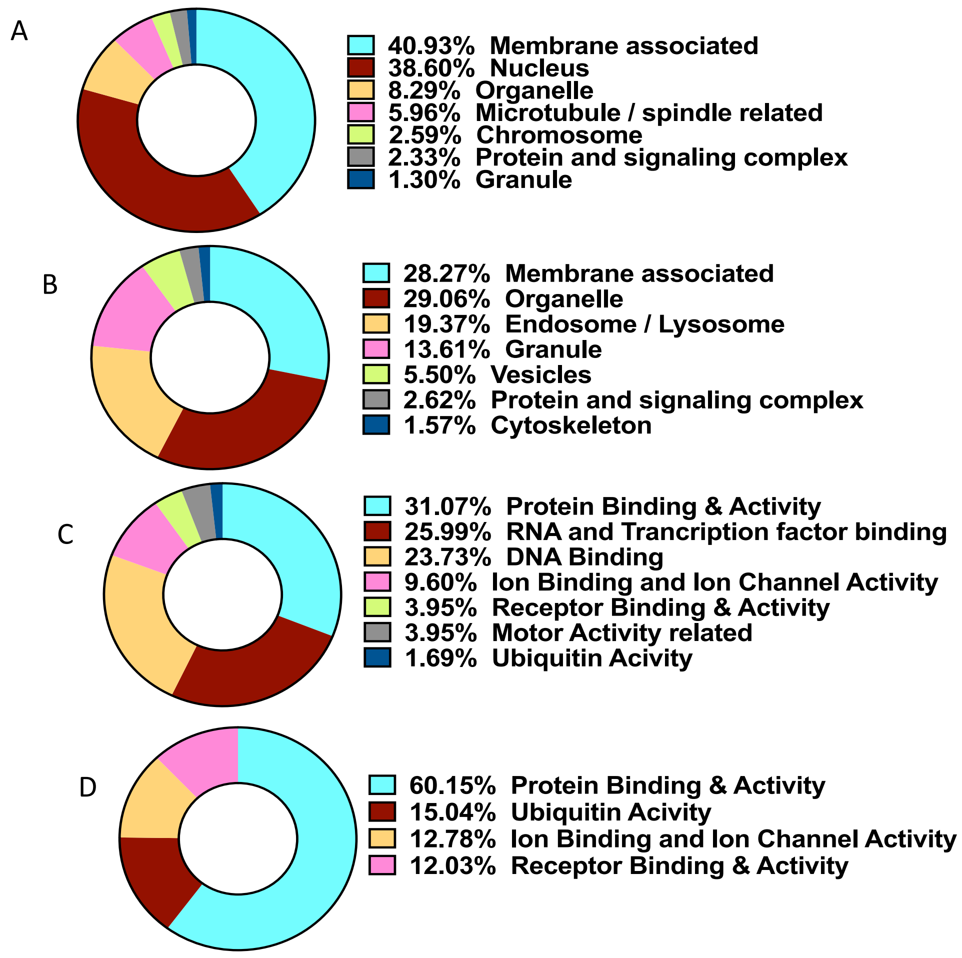

FIGURE S5

Fig. S5. RNA sequencing analysis revealed altered Gene Ontology terms in miR-30e expressing cells compared to control. (A B) Gene Ontology (GO) Enrichment analysis for the differentially expressed genes with p-value < 0.05. The top altered GO terms were represented as Circular plots showing the altered terms for each specific GO term based on Cellular Component (CC) for downregulated (**A**) and upregulated genes (**B**). **(C, D)** The top altered GO terms were represented as Circular plots showing the altered terms for Molecular Function (MF) for downregulated (**C**) and upregulated genes **(D)**.

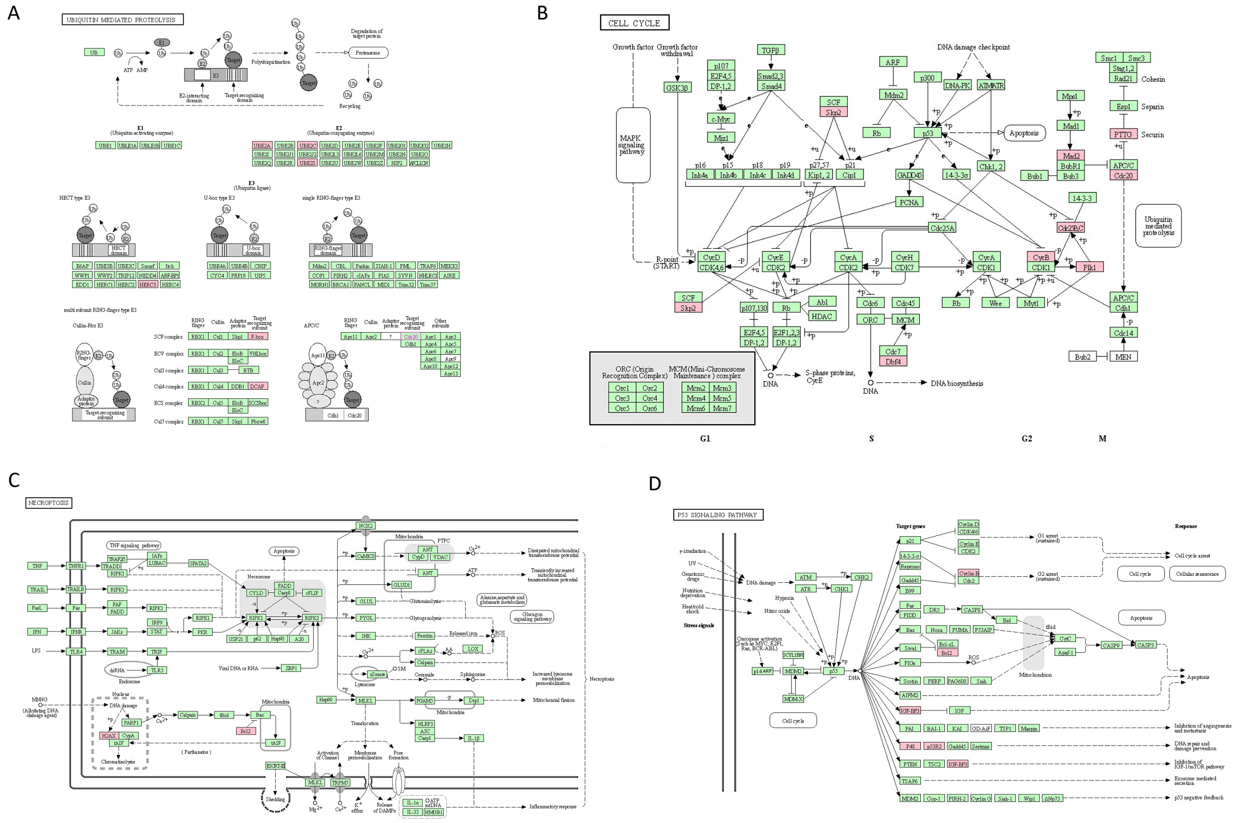

FIGURE S6

Fig. S6. miR-30e expression revealed top KEGG pathways that were enriched for the downregulated genes. KEGG Pathway analysis for the downregulated genes with p-value < 0.05. The top altered pathways for the downregulated genes included Ubiquitin mediated proteolysis **[A]**, Cell cycle **[B]**, Necroptosis **[C]** and p53 signaling pathway **[D]**.

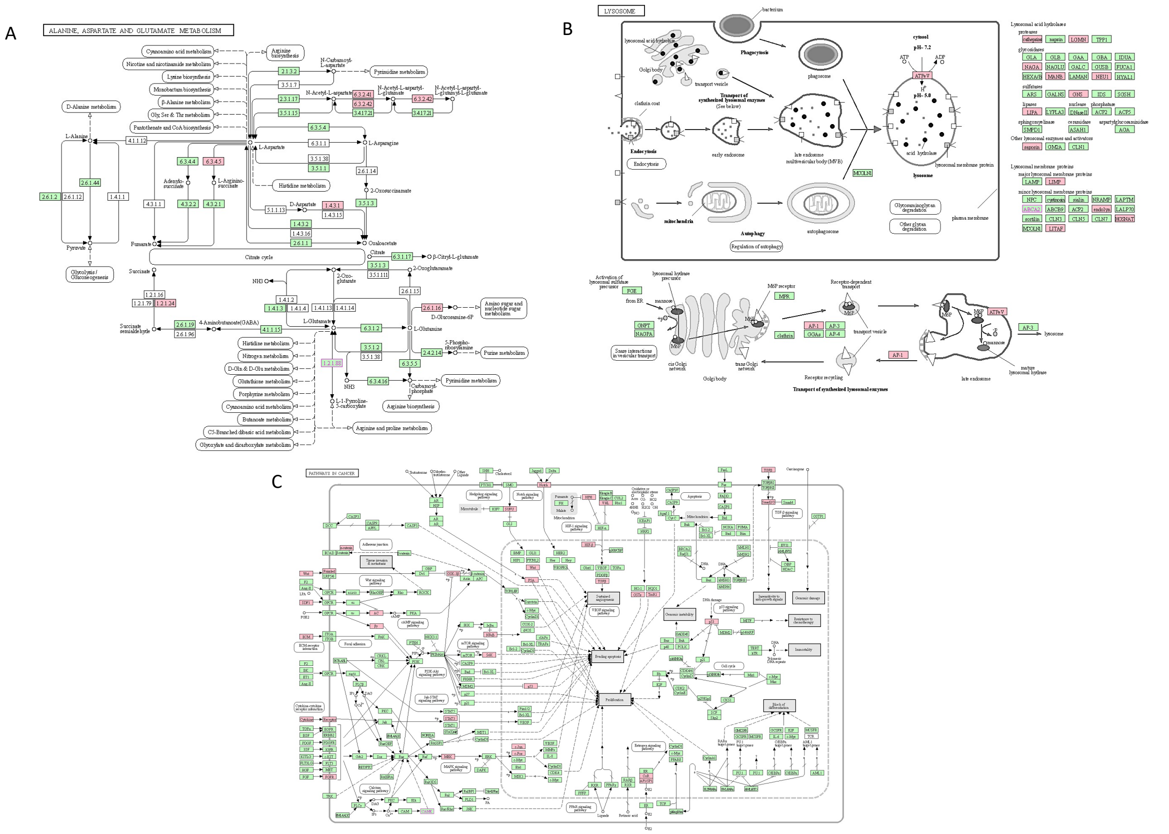

FIGURE S7

Fig. S7. miR-30e expression revealed top KEGG pathways that were enriched for the upregulated genes. KEGG Pathway analysis for the upregulated genes with p-value < 0.05 using Enrichr software. The top altered pathways for the upregulated genes included metabolic pathway **[A]**, Lysosome **[B]** and Pathways in Cancer **[C]**.

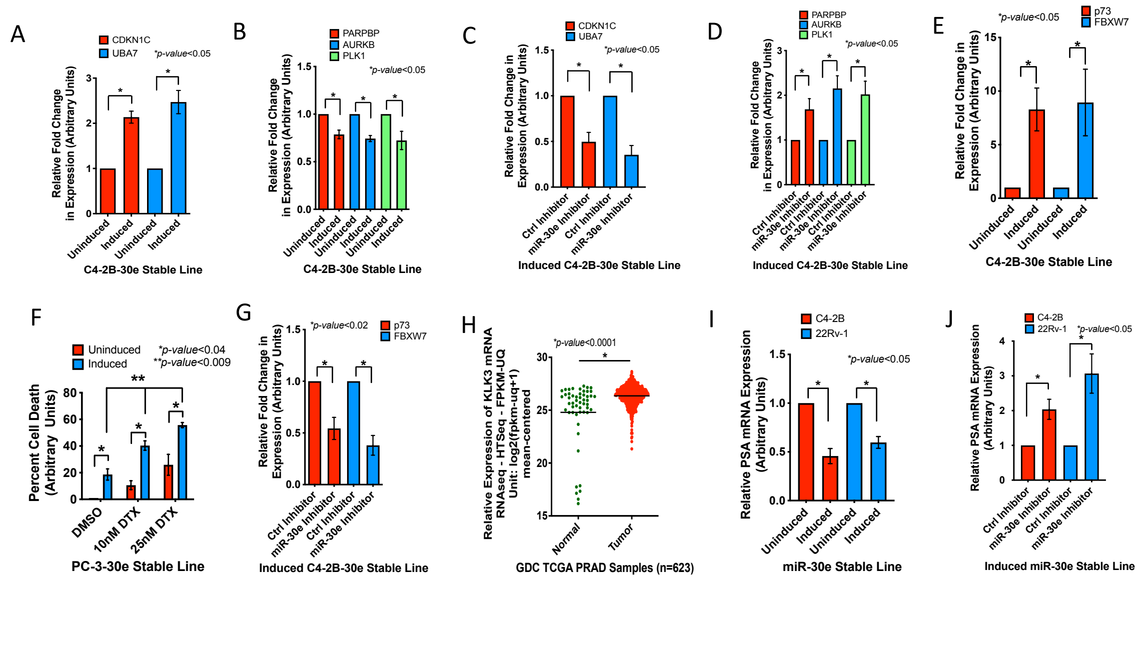

FIGURE S8

Fig. S8: miR-30e overexpression revealed decreased expression of genes involved in cell cycle progression, apoptosis, ubiquitination and AR signaling. (A, B) Validation of expression of selected genes upon overexpression of miR-30e in the induced C4-2B-30e cells compared to uninduced controls. qRT-PCR analysis revealed significantly increased expression of CDKN1C, UBA7 (**A**), and significantly decreased expression of PARPBP, AURKB and PLK1(**B**) upon overexpression of miR-30e in the induced C4-2B-30e cells compared to uninduced controls. (**C, D**) Confirmation of the expression results upon knockdown of miR-30e in the induced C4-2B-30e transfected with miR-30e inhibitor or control inhibitor. qRT-PCR analysis revealed significantly decreased expression of CDKN1C, UBA7 (**C**) and significantly increased expression of PARPBP, AURKB and PLK1 (**D**) in the inhibitor treated induced C4-2B-30e cells. (**E**) qRT-PCR analysis of FBXW7 and p73 expression showing upregulation of both mRNAs upon induction of miR-30e expression in C4-2B-30e cells compared to uninduced control cells. (**F**) Upregulation of FBXW7 and p73 mRNAs were also noted in 22Rv-1 xenograft tumors from the doxycycline induced group compared to the uninduced group. (**G**) Inhibition of miR-30 expression in induced C4-2B-30e cells using miR-30 inhibitor showed decreased expression of FBXW7 and p73 in these cells compared to the control cells. (**H)** We examined expression of KLK3 (PSA), a transcriptional target of AR. Clinical significance of KLK3 in the TCGA PRAD dataset analysis (n=623) revealed significantly upregulated expression of KLK3 in the PCa tumor tissues compared to normal tissues. **(I)** qRT-PCR analysis revealed decreased expression of KLK3 mRNA in the induced C4-2B-30e and 22Rv-1-30e cells compared to the uninduced control. (**J)** qRT-PCR analysis revealed increased expression of KLK3 mRNA in the induced C4-2B-30e and 22Rv-1-30e cells transfected with the miR-30e inhibitor compared to the control inhibitor. All data show the mean ± SD of at least 3 independent experiments.

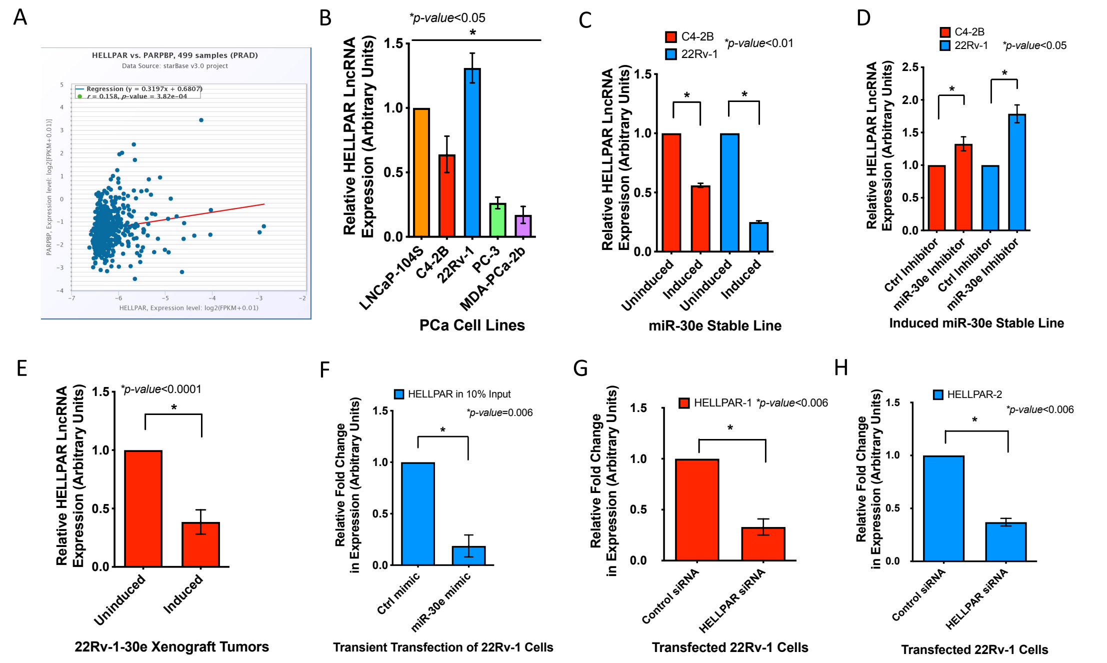

FIGURE S9

Fig. S9: A reciprocal functional relationship between miR-30e and *HELLPAR*. All data show the mean ± SD of at least 3 independent experiments. A second set of primers were used to confirm the qRT-PCR data. **(A)** ENCORI database analysis demonstrated a positive correlation of expression between *HELLPAR* lncRNA and PARPBP mRNA. **(B)** Endogenous expression of *HELLPAR* using primer set 2 in PCa cell lines by qRT-PCR analysis. Average fold change in expression of *HELLPAR* revealed highest expression in the 22Rv-1 PCa cells. Raw data was normalized to the expression of TBP housekeeping gene. **(C)** qRT-PCR analysis of miR-30e expressing induced C4-2B-30e and induced 22Rv-1-30e cells showing decreased expression of *HELLPAR* compared to the uninduced controls. **(D)** qRT-PCR analysis of miR-30e knockdown in the induced C4-2B-30e and induced 22Rv-1-30e cells revealed increased expression of *HELLPAR* lncRNA using primer set 2 compared to the control knockdown. *$p<0.04$. **(E)** qRT-PCR analysis of miR-30e expressing induced xenograft tumor tissues revealed decreased expression of *HELLPAR* lncRNA using primer pair 2 compared to uninduced control. **(F)** RNA immunoprecipitation of 22Rv-1 cells transfected with biotinylated miR-30e mimic or control mimic. qRT-PCR analysis of 10% of input RNA showed decreased *HELLPAR* lncRNA expression in the induced C4-2B-30e cells compared to the uninduced control. *$p=0.006$. **(G-H)** qRT-PCR analysis of *HELLPAR* expression detected by primer pairs 1 and 2 confirmed knockdown of *HELLPAR* in the wild-type 22Rv-1 cells transfected with *HELLPAR* siRNA pool compared to control siRNAs.
